# Impact of TG4010 Vaccine on Health-Related Quality of Life in Advanced Non-Small-Cell Lung Cancer: Results of a Phase IIB Clinical Trial

**DOI:** 10.1371/journal.pone.0132568

**Published:** 2015-07-24

**Authors:** Christine Rotonda, Amélie Anota, Mariette Mercier, Bérangère Bastien, Gisèle Lacoste, Jean-Marc Limacher, Elisabeth Quoix, Franck Bonnetain

**Affiliations:** 1 National Quality of Life in Oncology Platform, Nancy, France; 2 INSERM, CIC-EC CIE6, Nancy, France; 3 CHU Nancy, Clinical Epidemiology and Evaluation Department, Nancy, France; 4 Université de Franche-Comté, EA 3181, Besançon, France; 5 CHU Besançon, Methodological and quality of life unit in oncology, Besançon, France; 6 Transgene SA, Illkirch, France; 7 Hôpitaux Universitaires de Strasbourg, Strasbourg, France; Catalan Institute of Oncology, SPAIN

## Abstract

**Background:**

This study describes the effect of TG4010 vaccine on Health related Quality of Life (HRQOL) in patients with stage IIIb and IV non–small-cell lung cancer (NSCLC).

**Methods:**

148 patients with advanced NSCLC expressing MUC1 were randomly assigned to receive TG4010 plus chemotherapy or chemotherapy alone. HRQOL was assessed with the Functional Assessment of Cancer Therapy-Lung (FACT-L) at baseline and every 6 weeks until disease progression. Time until definitive deterioration (TUDD) of the four well-being dimensions of the FACT-L physical (PWB), functional (FWB), emotional (EWB) and social well-being (SWB) and the Lung Cancer Subscale (LCS) domains were analyzed for a 5-point minimal clinically important difference.

**Results:**

No difference of TUDD of HRQOL has been found between treatment arms. No prognostic factors have been found to have a significant impact on the TUDD of PWB, SWB and LCS domains. The gender, the performance status and the smoking habits seemed to be associated with a shorter TUDD of EWB domain. The smokers and the former smokers seemed to present a shorter TUDD of FWB domain.

**Conclusion:**

This study suggests that adding therapeutic vaccination with TG4010 to standard chemotherapy in patients with advanced NSCLC is associated with a similar evolution in HRQOL compared to chemotherapy alone.

## Introduction

Lung cancer is the most common cancer worldwide and the leading cause of cancer death [1].

About 85% to 90% of lung cancers are non-small cell lung cancer (NSCLC) and of these about 75% have locally advanced or disseminated disease at the time of diagnosis. The standard treatment for advanced NSCLC is chemotherapy [2,3]. Traditional chemotherapy regimens have shown real but limited activity in this setting, therefore new strategies are being explored for lung cancer treatment, including targeted active immunotherapies [4,5]. Recent research suggests that the use of therapeutic cancer vaccines may improve overall survival (OS), with reduced toxicity compared with conventional chemotherapy [6]. It is unanimously accepted that the goal of therapy for advanced NSCLC patients is prolongation of OS without negative impact on Health related Quality of Life (HRQOL) and ideally with an improvement of it [7]. Moreover, even when there is no apparent advantage in OS for a new treatment, a positive effect on HRQOL can be seen as a real improvement [8]. The literature also shows the importance of HRQOL as an important prognostic factor of OS in various cancer sites and particularly in lung cancer [9–11]. Therefore, clinical trials including OS in the endpoints are now incorporating symptom scores and HRQOL outcomes in their designs. The potential benefits of palliative chemotherapy on HRQOL have been investigated and demonstrated for several agents in lung cancer trials [12].

A phase IIb multicentric controlled trial was developed to assess TG4010 –an active targeted immunotherapy based on a viral MVA vector which codes for MUC1 tumor-associated antigen and interleukine 2 –in combination with first-line chemotherapy in patients with advanced NSCLC. The primary objective of the study was to show that the addition of TG4010 to chemotherapy improved the progression-free survival (PFS) at 6 months. Both OS and HRQOL were assessed as secondary objectives. The study achieved its primary endpoint on the whole study population and in an exploratory analysis put in evidence a significant benefit on several parameters including OS in a large subgroup of 101 patients defined by pre-treatment normal levels of CD16+CD56+CD69+, a phenotype of activated Natural Killer (aNK) cells also called TrPAL (Triple Positive Activated Lymphocytes) [13]. We report here the results of the HRQOL analyses associated to this clinical trial. To our knowledge this is the first time that the impact of immunotherapy on HRQOL has been studied in the context of a combination with standard chemotherapy for NSCLC patients. The main objective was to describe prospectively HRQOL using the Functional Assessment of Cancer Therapy-Lung (FACT-L) questionnaire by treatment arm. Time until definitive deterioration (TUDD) of the FACT-L domains was defined as a modality of longitudinal HRQOL analysis [14].

## Materials and Methods

### Study population and treatments

The assessment of HRQOL was a secondary endpoint of a multicentric open label randomized phase IIb trial performed to test the hypothesis that the addition of TG4010 to first line chemotherapy would improve the outcome in patients with advanced NSCLC (NCT00415818). The main eligibility patients’ criteria were patients aged 18 years or older with a confirmed stage IIIb or IV NSCLC, chemotherapy naive for this stage of the disease, expressing MUC1, with at least one lesion measurable by CT-scan according to World Health Organization, a performance status (PS) of 0 or 1 and a life expectancy of at least 4 months. Patients were excluded if they had received prior systemic therapy for advanced stage NSCLC, or if they had concomitant brain metastases (unless successfully treated) or history of another malignancy within the past 5 years, apart from basal-cell carcinoma of the skin or intraepithelial carcinoma of the cervix. The study obtained approval from national health agencies and ethics committees in relevant countries and was done in accordance with the Helsinki Declaration, the International Conference of Harmonization Good Clinical Practice guidelines, and local regulatory requirements. Written informed consent was obtained from each patient. This study started almost 10 years ago and at this time it was not yet so common to register our studies, at least from the beginning. The authors confirm that all ongoing and related trials for this drug/intervention are registered.

The protocol for this trial and supporting CONSORT checklist are available as supporting information (see S1 Checklist and S1 Protocol). List of Ethics Committees is also available in supporting information (see S1 Authorization).

Patients were allocated by a 1:1 randomization (minimization procedure stratified by centre (27 centres of France, Poland, Hungary and Germany), PS (0 vs. 1) and disease stage (IIIb vs. IV)) in one of the two following treatment regimens: in arm 1, patients received chemotherapy (gemcitabine + cisplatin up to 6 cycles) combined with TG4010 subcutaneously at the dose of 10^8^ pfu once per week for 6 weeks then once every 3 weeks up to progression (combination therapy arm), in arm 2, patients received the same chemotherapy alone (control arm).

### Sample size calculation

The primary endpoint of this study was 6-month PFS in combination therapy arm. The study was designed as a one-stage, phase IIB trial according to Fleming [15] with an inactivity cut-off chosen as 30% and activity cut-off as 50%. Therefore, the hypotheses of interest were 30% or less patients who were progression free at 6 months (null hypothesis) versus 50% or more patients who were progression free at 6 months. The type I error rate and the type II error rate were both set to 5%, leading to a total sample size of 140 patients. To reject the null hypothesis (6-month PFS ≤30% with combination therapy), at least 40% of patients in the TG4010 group had to be progression free at 6 months.

### Health related Quality of life assessment

HRQOL was evaluated using the FACT-L questionnaire at inclusion, every 6 weeks until the end of study (disease progression) and at the end of study visit. The questionnaire had to be fulfilled by the patient at the hospital (during the treatment or the end of treatment visit). The FACT-L is composed by the FACT-General with the specific module of the lung cancer. It contains 36 items in order to measure 4 well-being dimensions: physical well-being (PWB), social well-being (SWB), emotional well-being (EWB) and functional well-being (FWB) and the Lung Cancer Subscale (LCS) [16]. Three global scores are calculated: the FACT-G global score, the FACT-L global score and the Trial Outcome Index (TOI) which is the sum of PWB, FWB and LCS scores. Scores were linearly adjusted to range scores from 0 to 100. A high score corresponded to a high HRQOL level. The HRQOL targeted dimensions were the four well-being domains (PWB, SWB, EWB and FWB) and the LCS.

### Statistical analysis

Patient’s socio-demographic and clinical characteristics at baseline were described. The percentage of available questionnaires was reported at each assessment. Patients with HRQOL questionnaire fully completed at baseline and those with at least one missing item at baseline were compared according to baseline characteristics and treatment arm in order to detect non-random missing data profiles. Scores were calculated using the simple imputation method by the mean following the FACT-L scoring guidelines [16]. Scores were described by treatment arm at each measurement time using the mean and standard deviation (SD). Mean difference and 99% confidence interval (99%CI) were calculated at baseline. If the outcome data was normally distributed, Student t test was employed. However, if the outcome data was not normally distributed, Mann Whitney test was used instead.

The TUDD in a HRQOL score is defined as the interval between randomization and the date of the event, i.e. the date where the first significant deterioration in the HRQOL level of the patient was observed as compared to baseline HRQOL score, without any further significant improvement as compared to baseline HRQOL score [14]. Alive patients were censored at the time of the last follow-up if no definitive deterioration was observed before. Only patients with the baseline HRQOL score and at least one follow-up score or death were retained (modified intent to treat analysis). Each dimension was analysed. TUDD curves were calculated using the Kaplan-Meier estimation and were described using medians and 99%CI. As exploratory purpose only, TUDD curves were compared using the log-rank tests. The univariate Cox models were used to calculate the hazard ratio (HR) with a 99%CI. A subgroup analysis was also performed according to the level of aNK cells based on previous observed effect of aNK cells on OS. Variables investigated were gender (women vs. men), age (continuous variable), smoking habits (ceased vs. no vs. yes), PS (1 or more vs. 0), center (dichotomized according to the median of the distribution of patients included by center: ≤3 vs. >3) and the time to toxicity of grade 3–4 (time dependent variable). Variables with a univariate *P*-value ≤ 0.20 were eligible for the multivariate Cox analyses. Treatment arm and level of aNK cells subgroup (high vs. normal) were forced in multivariate Cox analyses. Their interaction was also introduced and exploratory subgroup analyses were performed according to pre-treatment level of aNK cells based on previous observed effect of aNK cells on OS [13]. Finally, the same variables were introduced in each multivariate model for all HRQoL dimensions.

Data were analysed using software SAS (Version 9.2, SAS Institute Inc, Cary, NC) and R [17]. All tests were two-sided and the type I error was set to 0.01 due to multiple comparisons (Bonferroni adjustment, 5 targeted dimensions of HRQOL).

## Results

### Study population

One hundred and forty eight patients with advanced stages of NSCLC were randomized: 74 in arm 1 and 74 arm 2. Details of patient’s selection criteria and results of the primary endpoint (i.e. “Progression Free Survival” at 6 months) have been reported elsewhere [13]. The mean age was 60.5 years (SD = 8.1). Fifty-four patients (73.0%) were male, 53 patients (76.8%) had normal level of aNK cells, 68 patients (91.9%) had a stage III cancer, 27 patients (36.5%) were smokers and 35 patients (47.3%) were former smokers (Table 1). Individual-level data are available as supporting information (see S1 Data).

**Table 1:** Baseline Characteristics of patients.

	All patients (n = 148)	Combination therapy group (n = 74)	Control group (n = 74)	P *
	Mean (SD)	Mean (SD)	Mean (SD)	
**Age**	59.41 (8.8)	58.36 (9.4)	60.48 (8.1)	0.145
**Sex**				0.854
Male	107 (72.3)	53 (71.6)	54 (73.0)	
Female	41 (27.7)	21 (28.4)	20 (27.0)	
**ECOG performance status**				0.604
0	40 (27.0)	20 (27.0)	20 (27.0)	
1	107 (72.3)	53 (71.6)	54 (73.0)	
2	1 (0.7)	1 (1.4)	0 (0.0)	
**Stage at baseline**				0.999
IIIB	12 (8.1)	6 (8.1)	6 (8.1)	
IV	136 (91.9)	68 (91.9)	68 (91.9)	
**Level of natural killer cells**				0.337
Normal	101 (73.2)	21 (30.4)	16 (23.2)	
High	37 (26.8)	48 (69.6)	53 (76.8)	
**Smoking habits**				0.772
No	22 (14.9)	10 (13.5)	12 (8.1)	
ceased	68 (46.0)	33 (44.6)	35 (47.3)	
Yes	58 (39.1)	31 (41.9)	27 (36.5)	

Data are n (%) unless otherwise specified

* T test for continuous variable and chi-square test or Fisher’s Exact Test for qualitative variables

Significance level set to 0.01 according to Bonferroni adjustment

### Missing data

**Fig 1:**
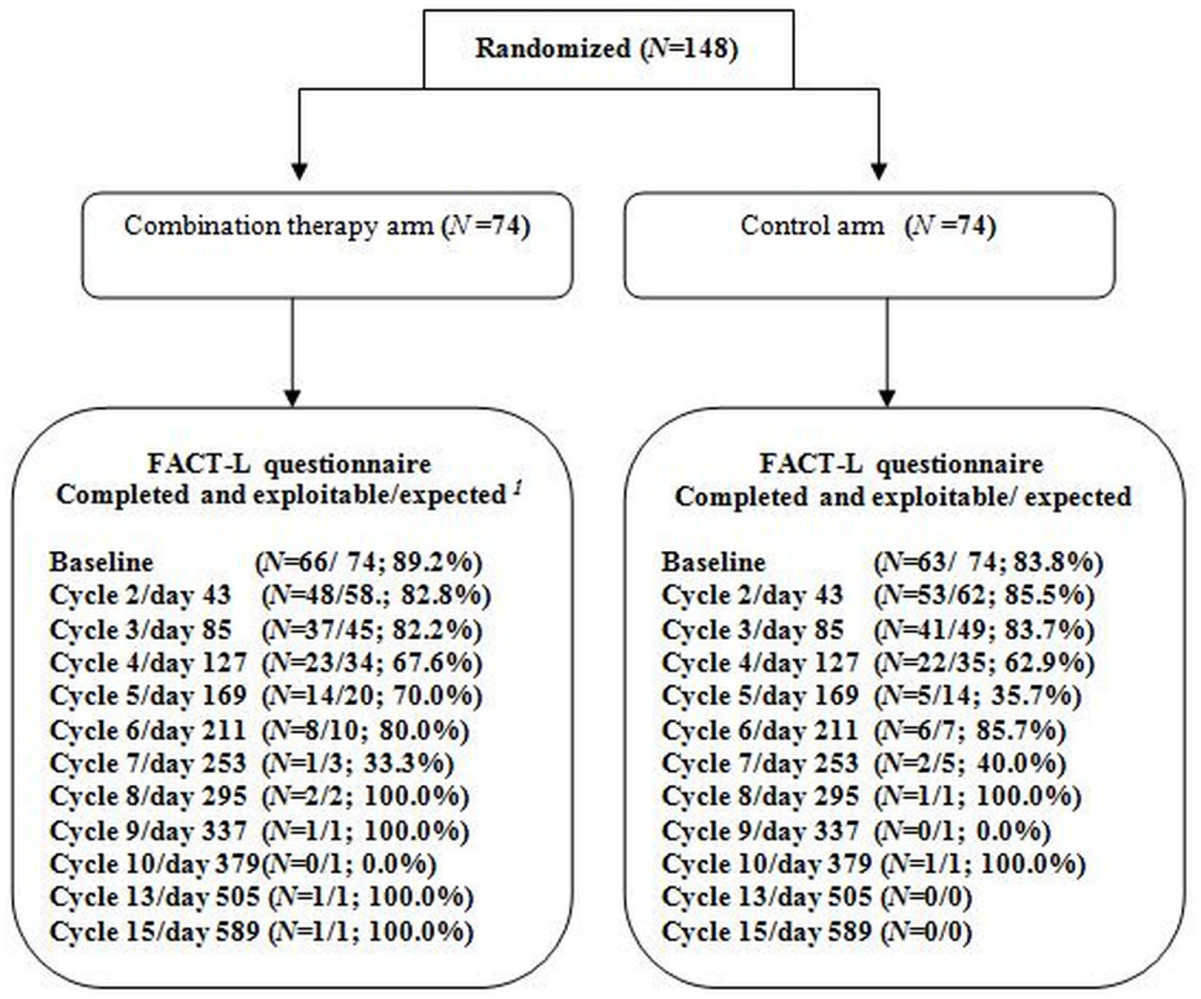
CONSORT Flow chart showing patients randomised in each group and the number of patients answering FACT-L questionnaire at baseline and follow-up. Patients still in the study time of the questionnaire completion.

At baseline, 129 patients (87.2%) completed the FACT-L questionnaire: 66 (89.2%) in arm 1 and 63 (83.8%) in arm 2. Fig 1 described the number and percentage of questionnaires completed at each measurement time according to expected completion (patients still in the study at the time of the questionnaire completion).

Among the completed and exploitable questionnaires (i.e. questionnaires with less than half of the items missing), less than 2% of the items were missing in all dimensions, at each measurement time, except for the SWB score for which around 10% of the items were missing. Nevertheless, the pattern of missing data was found to be random. Patients with at least one missing item at baseline and those with the entire questionnaire fulfilled were similar for all baseline socio-demographic characteristics.

### Description of FACT-L scores

Patients receiving standard treatment presented a better HRQOL at baseline than patients receiving the vaccine (Table 2). These differences were clinically significant with a MCID ≥ 5-point [18] for PWB (-6.86 [99%CI -15.97, 2.25]), EWB (-6.03 [99%CI -15.73, 3.66]) and for TOI (-5.55 [99%CI -13.33, 2.23]), FACT-G (99%CI -5.94 [-12.78, 0.91]) and FACT-L (99%CI -5.46 [-12.02, 1.09]) global scores but not statistically significant.

**Table 2:** Health Related Quality of Life at baseline according to type of treatment allocated by randomization.

	combination therapy arm (N = 74)		control arm (N = 74)		combination therapy arm—control arm	P (student t test)
	n	Mean (SD)	n	Mean (SD)	Mean difference [99%CI]	
**PWB score**	65	71.56 (21.79)	62	78.42 (17.03)	-6.86 [-15.97, 2.25]	0.110*
**SWB score**	66	76.28 (15.57)	62	80.75 (19.23)	-4.47 [-12.54, 3.60]	0.041*
**EWB score**	66	63.62 (23.19)	63	69.66 (18.56)	-6.03 [-15.73, 3.66]	0.222*
**FWB score**	66	53.92 (23.78)	62	60.45 (24.00)	-6.53 [-17.58, 4.52]	0.125
**LCS score**	67	67.16 (16.40)	63	71.03 (17.85)	-3.87 [-11.72, 3.99]	0.200
**TOI index**	65	64.26 (17.55)	61	69.81 (15.69)	-5.55 [-13.33, 2.23]	0.097*
**FACT-G global score**	65	66.37 (15.52)	61	72.31 (13.73)	-5.94 [-12.78, 0.91]	0.067*
**FACT-L global score**	65	66.48 (14.74)	61	71.95 (13.28)	-5.46 [-12.02, 1.09]	0.068*

CI: confidence interval

PWB: Physical Well-Being dimension; SWB: Social Well-Being; EWB: Emotional Well-Being, FWB: Functional Well-Being; LCS: Lung Cancer Subscale; TOI: Trial Outcome Index

Significance level set to 0.01 according to Bonferroni adjustment

*: Mann-Whitney test

### Longitudinal analysis of health-related Quality of Life

#### TUDD of Physical Well-Being score with a 5-point MCID or death

116 patients are retained (58 patients per arm) with at least the baseline PWB score and one follow-up PWB score and/or who died during the study.

**Fig 2:**
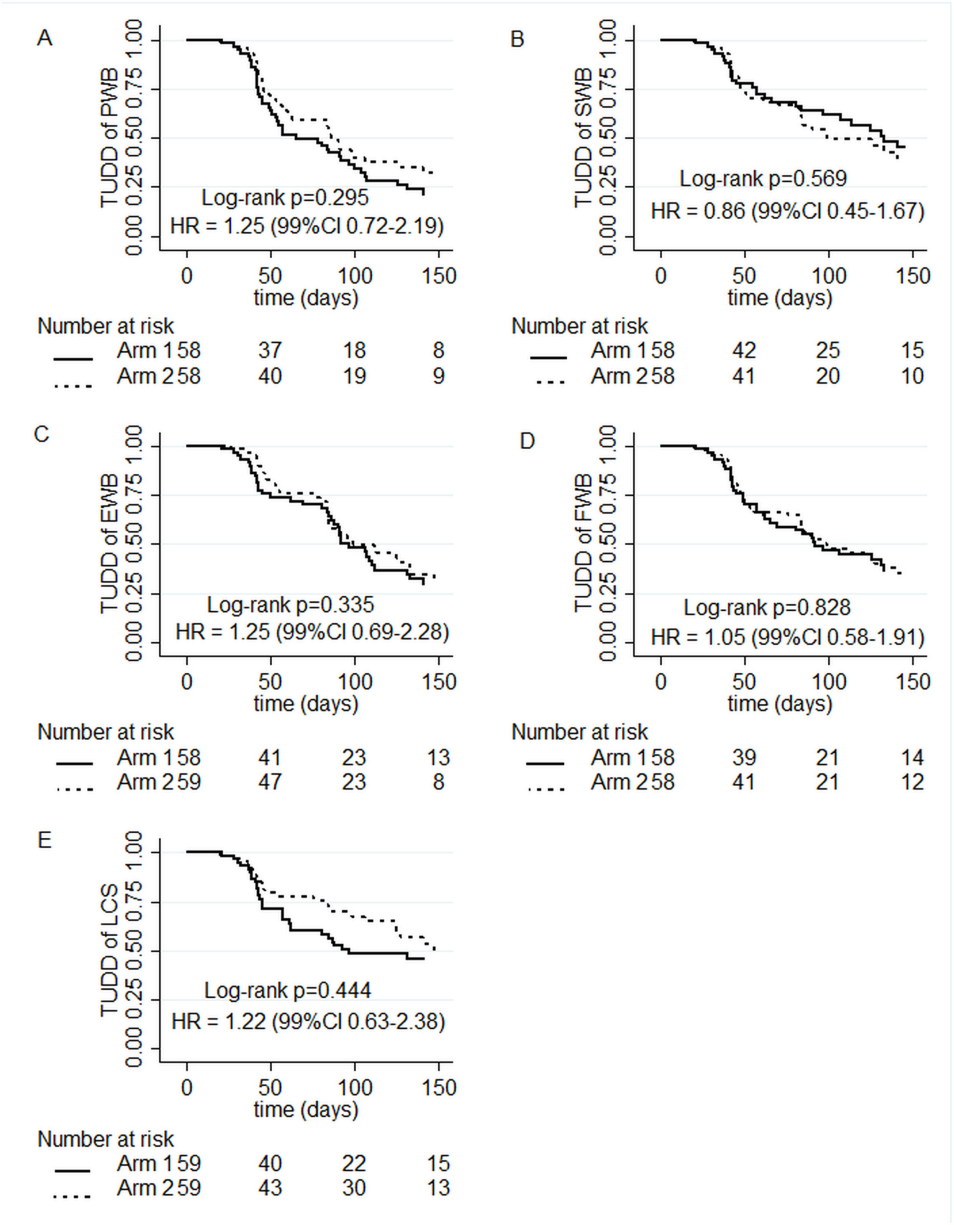
Time to a five-point definitive deterioration in Health related Quality of Life score or death: (A) Physical Well-Being dimension (PWB); (B) Emotional Well-Being dimension (EWB); (C) Social Well-Being dimension (SWB); (D) Functional Well-Being dimension (FWB); (E) Lung Cancer Subscale (LCS). Arm 1: combination therapy arm; Arm 2: control arm.

In arm 1 and arm 2 respectively, 46 and 41 patients experienced a definitive deterioration of PWB score of 5 points at least or death (Fig 2, panel A), among the 116 patients retained (58 patients per arm). The median TUDD was 65 days (99%CI 50–107) in arm 1 and 86 days (99%CI 60–201) in arm 2 (log-rank *P =* 0.295). The Univariate Cox HR of arm 1 vs. arm 2 was 1.25 (99%CI 0.72–2.19).

#### TUDD of Social-Well-Being score of 5-point or death

116 patients are retained (58 patients in each arm) with at least the baseline SWB score and one follow-up SWB score and/or who died during the study.

In arm 1 and arm 2 respectively, 30 and 32 patients experienced a definitive deterioration of SWB score of 5 points at least or death (Fig 2, panel B) among the 116 patients included (58 patients per arm). The median TUDD was 133 days (99%CI 84-NA) in arm 1 and 99 days (99%CI 82-NA) in arm 2 (log-rank *P =* 0.569). The Univariate Cox HR was 0.86 (99%CI 0.45–1.67).

#### TUDD of Emotional Well-Being score with a 5-point MCID or death

117 patients are retained (58 in arm 1 and 59 in arm 2) with at least the baseline EWB score and one follow-up EWB score and/or who died during the study.

In arm 1 and arm 2 respectively, 41 and 34 patients experienced a definitive deterioration of EWB score of 5 points at least or death (Fig 2, panel C), among the 117 patients included (58 in arm 1 and 59 in arm 2). The median TUDD was 97 days (99%CI 85–169) in arm 1 and 99 days (99%CI 84-NA) in arm 2 (log-rank *P =* 0.335). The Univariate Cox HR was 1.25 (99%CI 0.69–2.28).

#### TUDD of Functional Well-Being score with a 5-point MCID or death

116 patients are retained (58 patients in each arm) with at least the baseline FWB score and one follow-up FWB score and/or who died during the study.

In arm 1 and arm 2 respectively, 38 and 37 patients experienced a definitive deterioration of FWB score of 5 points at least or death (Fig 2, panel D), among the 116 patients included (58 patients per arm). The median TUDD was 92 days (99%CI 62-NA) in arm 1 and 99 days (99%CI 76-NA) in arm 2 (log-rank *P =* 0.828). The Univariate Cox HR was 1.05 (99%CI 0.58–1.91).

#### TUDD of Lung Cancer Subscale score with a 5-point MCID or death

118 patients are retained (59 patients per arm) with at least the baseline LCS score and one follow-up LCS score and/or who died during the study.

In arm 1 and arm 2 respectively, 32 and 28 patients experienced a definitive deterioration of LCS score of 5 points at least or death (Fig 2, panel E), among the 118 patients included (59 patients per arm). The median TUDD was 97 days (99%CI 61-NA) for arm 1 and 147 days (99%CI 125-NA) for arm 2 (log-rank *P =* 0.444). The Univariate Cox HR was 1.22 (99%CI 0.63–2.38).

#### Multivariate Cox analyses

Table 3 presents univariate and multivariate Cox analyses of TUDD.

**Table 3:** Univariate and multivariate Cox analyses for a 5-point definitive deterioration in Health Related Quality of Life scores or death.

	Univariate Cox analysis			Multivariate Cox analysis		
	n (events)	HR (99%CI)	P-value	n (events)	HR (99%CI)	P-value
**PWB**	116 (87)			110 (81)		
combination therapy arm vs. control arm^1^		1.25 (0.80–2.19)	0.295		1.36 (0.67–2.76)	0.260
sex: women vs. men		1.00 (0.54–1.85)	0.999		0.95 (0.49–1.85)	0.834
age ^2^		1.00 (0.97–1.04)	0.827			
smoking habits			0.450			0.581
Yes vs. No		1.51 (0.64–3.56)	0.219		1.41 (0.58–3.39)	0.318
Ceased vs. No		1.34 (0.59–3.07)	0.360		1.33 (0.56–3.14)	0.399
aNK cells: high vs. normal^1^		1.33 (0.71–2.52)	0.243		1.42 (0.56–3.61)	0.326
center: ≤3 vs. >3		1.13 (0.88–1.98)	0.565			
performance status: 1 or more vs. 0		0.79 (0.42–1.47)	0.319		0.73 (0.37–1.46)	0.241
time to toxicity grade 3–4		1.16 (0.74–1.83)	0.513			
treatment arm * aNK cells ^1^		0.95 (0.27–3.56)	0.912		0.82 (0.23–3.03)	0.704
**SWB**	116 (62)			110 (59)		
combination therapy arm vs. control arm ^1^		0.86 (0.45–1.67)	0.569		0.79 (0.40–1.59)	0.470
sex: women vs. men		0.85 (0.41–1.77)	0.580		1.03 (0.48–2.22)	0.926
age ^2^		0.99 (0.95–1.03)	0.379			
smoking habits			0.125			0.081
Yes vs. No		2.01 (0.74–5.48)	0.071		2.13 (0.76–5.98)	0.062
Ceased vs. No		1.32 (0.49–3.60)	0.470		1.24 (0.43–3.58)	0.608
aNK cells: high vs. normal^1^		0.87 (0.40–1.85)	0.624		0.90 (0.42–1.92)	0.793
center: ≤3 vs. >3		1.45 (0.61–2.29)	0.170			
performance status: 1 or more vs. 0		1.66 (0.75–3.65)	0.098		1.75 (0.76–4.03)	0.088
time to toxicity grade 3–4		1.06 (0.62–1.80)	0.848			
treatment arm * aNK cells ^1^		1.05 (0.23–4.77)	0.936		1.00 (0.42–1.92)	0.994
**EWB**	117 (75)			111 (70)		
combination therapy arm vs. control arm 1		1.25 (0.69–2.28)	0.335		1.20 (056–2.59)	0.532
sex: women vs. men		0.47 (0.22–0.99)	0.009		0.43 (0.19–0.97)	0.008
age ^2^		0.98 (0.95–1.02)	0.161			
smoking habits			0.013			0.021
Yes vs. No		3.19 (1.07–9.47)	0.006		2.88 (0.95–8.71)	0.014
Ceased vs. No		2.93 (1.01–8.55)	0.009		2.62 (0.87–7.86)	0.024
aNK cells: high vs. normal^1^		1.47 (0.76–2.85)	0.129		1.79 (0.62–5.15)	0.158
center: ≤3 vs. >3		1.02 (0.56–1.87)	0.621			
performance status: 1 or more vs. 0		1.96 (0.92–4.13)	0.021		2.17 (0.96–4.93)	0.010
time to toxicity grade 3–4		1.05 (0.64–1.71)	0.856			
treatment arm * aNK cells ^1^		1.45 (0.37–5.63)	0.478		1.22 (0.31–4.79)	0.704
**FWB**	116 (63)			110 (69)		
combination therapy arm vs. control arm ^1^		1.05 (0.58–1.91)	0.828		0.81 (0.37–1.74)	0.469
sex: women vs. men		0.68 (0.34–1.36)	0.149		0.65 (0.31–1.37)	0.137
age ^2^		1.00 (0.97–1.04)	0.804			
smoking habits			0.027			0.042
Yes vs. No		2.73 (1.01–7.37)	0.009		2.57 (0.93–7.09)	0.016
Ceased vs. No		2.34 (0.88–6.24)	0.025		2.14 (0.76–6.05)	0.059
aNK cells cells: high vs. normal^1^		1.26 (0.65–2.46)	0.368		0.96 (0.35–2.65)	0.921
center: ≤3 vs. >3		1.06 (0.58–1.94)	0.795			
performance status: 1 or more vs. 0		0.99 (0.51–1.93)	0.987		1.01 (0.47–2.13)	0.986
time to toxicity grade 3–4		1.09 (0.66–1.78)	0.746			
treatment arm * aNK cells ^1^		2.27 (0.57–9.00)	0.124		2.03 (0.51–8.10)	0.189
**LCS**	118 (60)			111 (56)		
combination therapy arm vs. control arm ^1^		1.22 (0.63–2.38)	0.444		1.11 (0.46–2.66)	0.760
sex: women vs. men		0.71 (0.32–1.55)	0.258		0.76 (0.32–1.77)	0.401
age ^2^		1.01 (0.97–1.05)	0.535			
smoking habits			0.815			0.942
Yes vs. No		1.22 (0.46–3.26)	0.601		1.05 (0.38–2.94)	0.897
Ceased vs. No		1.04 (0.40–2.72)	0.908		0.95 (0.34–2.62)	0.897
aNK cells: high vs. normal^1^		1.36 (0.66–2.82)	0.279		1.13 (0.37–3.40)	0.777
center: ≤3 vs. >3		1.95 (0.97–3.92)	0.014			
performance status: 1 or more vs. 0		1.47 (0.65–3.29)	0.222		1.34 (0.56–3.20)	0.384
time to toxicity grade 3–4		1.46 (0.83–2.55)	0.192			
treatment arm * aNK cells ^1^		1.50 (0.34–6.55)	0.477		1.48 (0.33–6.73)	0.506

PWB: Physical Well-Being dimension; SWB: Social Well-Being; EWB: Emotional Well-Being, FWB: Functional Well-Being; LCS: Lung Cancer Subscale, aNK cells: activated Natural Killer cells.

^1^ variable forced in the multivariate analysis

^2^ continuous variable

*: interaction

#### Significance level set to 0.01 according to Bonferroni adjustment

Cox multivariate analyses showed that no variables had a significant impact on the TUDD of PWB, SWB and LCS domains. However the gender (male) (P = 0.009) was associated with a shorter TUDD of EWB domain. The smokers and the former smokers tended to present a shorter TUDD of FWB domain, but not statistically significant. The interaction test between treatment arm and the levels of aNK cells was not significant (P = 0.704; 0,994; 0.704; 0.189 and 0.506 respectively for PWB, SWB, EWB, FWB and LCS domains).

TUDD curves estimations according to the level of aNK cells did not reflect a significant effect of the level of aNK cells on the TUDD (S1 and S2 Figs). However, patients with a high level of pre-treatment aNK cells seemed to have a shorter TUDD than those with normal level of aNK cells for PWB (HR arm 1 vs. arm 2: 1.39 (0.50–4.30) for high level of aNK cells vs. 1.29 (0.65–2.57) for normal level of aNK cells), EWB (1.58 (0.52–4.77) vs. 1.15 (0.53–2.47)), FWB (2.23 (0.65–7.65) vs. 0.83 (0.39–1.79)) and LCS domains (1.82 (0.54–6.17) vs. 1.11 (0.47–2.61)), but all these trends were not statistically significant (Table 4).

**Table 4:** Results of the Kaplan-Meier estimation of the time until definitive deterioration curves according the level of activated Natural Killer (aNK) cells.

	patients with normal level of aNK cells				patients with high level of aNK cells			
	n (events)	median—days (99%CI)	P log-rank	HR combination therapy arm vs. control arm (99%CI)	n (events)	median—days (99%CI)	P log-rank	HR combination therapy arm vs. control arm (99%CI)
**PWB**								
combination therapy arm	37 (30)	81 (54–169)	0.355	1.29 (0.65–2.57)	18 (13)	65 (39-NA)	0.439	1.39 (0.50–4.30)
control arm	42 (27)	91 (62-NA)			13 (11)	85 (50-NA)		
**SWB**								
combination therapy arm	37 (20)	133 (81-NA)	0.448	0.79 (0.35–1.77)	18 (8)	131 (45-NA)	0.715	0.83 (0.23–3.03)
control arm	42 (23)	99 (61-NA)			13 (8)	141 (82-NA)		
**EWB**								
combination therapy arm	37 (25)	107 (90-NA)	0.644	1.15 (0.53–2.47)	18 (14)	86 (39-NA)	0.276	1.58 (0.52–4.77)
control arm	43 (22)	125 (85-NA)			13 (9)	96 (84-NA)		
**FWB**								
combination therapy arm	37 (22)	126 (65-NA)	0.536	0.83 (0.39–1.79)	18 (13)	90 (44-NA)	0.086	2.23 (0.65–7.65)
control arm	42 (25)	98 (76-NA)			13 (9)	141 (84-NA)		
**LCS**								
combination therapy arm	37 (19)	166 (61-NA)	0.760	1.11 (0.47–2.61)	18 (11)	88 (39-NA)	0.202	1.82 (0.54–6.17)
control arm	43 (18)	183 (98-NA)			13 (8)	141 (125-NA)		

PWB: Physical Well-Being dimension; SWB: Social Well-Being; EWB: Emotional Well-Being, FWB: Functional Well-Being; LCS: Lung Cancer Subscale

Significance level set to 0.01 according to Bonferroni adjustment

## Discussion

The role of therapeutic vaccines in advanced cancer patients would be like with chemotherapy to increase the time to progression and to increase survival while preserving HRQOL. The results previously reported presented the efficacy and safety data of the therapeutic vaccine TG4010 combined with chemotherapy as a first line treatment for patients advanced NSCLC. PFS at 6 months for vaccinated patients was found to be 43% and for the patients on standard chemotherapy was 35% [13]. A significant benefit of survival was observed in the subgroup of patients with normal level of pre-treatment aNK cells, although this finding was based on an unplanned exploratory analysis. According two meta-analyses, a gain in HRQOL has been observed in 50% of trials even if no benefit in term of OS was showed [19,20]. In our study, missing HRQOL data were low except for SWB domain. Concerning this domain, some patients could have chosen to not answer especially questions about sexual activities. Our results showed no statistical difference of TUDD of HRQOL score between the two evaluated therapeutic strategies supporting the good tolerance of TG4010 when added to chemotherapy in this patient population. Only the gender "men" was significantly associated with a shorter TUDD for EWB domain.

Few studies evaluated HRQOL in NSCLC patients treated by therapeutic vaccination and they found benefits to patient HRQOL by additional vaccination [21,22]. Butt et al. showed that HRQOL was maintained longer in patients who received L-BLP25 vaccine and best supportive care (BSC) compared to patients who received BSC alone. HRQOL results obtained in randomized trials using either L-BLP25 or TG4010 suggest that therapeutic vaccine have no negative impact on HRQOL. Further work would be necessary to confirm these findings and incorporation of HRQOL in pivotal phase III trials with therapeutic vaccines is essential. This applies both to trials testing a vaccine in addition to BSC or combined with standard of care.

In the original trial, pre-treatment aNK cells seemed to be a biomarker to target patients appropriate for a treatment with TG4010 [13]. Indeed, an improved clinical outcome for patients with a normal level of aNK cells was shown with a 6-month increase in median OS (17.1 months in the vaccine group versus 11.3 months in the control group) [13]. HRQOL analyses did not differ strikingly according to the level of pre-treatment aNK cells. However, when treated by TG4010 with a normal level of aNK cells tended to present a longer TUDD than patients with a high level of aNK cells. Nevertheless, we failed to demonstrate a statistically significant interaction between treatment arms and level of aNK cells, probably due to a lack of statistical power. Moreover, there were more patients with no follow-up measure in the subgroup of high level of aNK cells: it is suspected that these patients had a lower general health status than those with normal level of aNK cells and were unable to complete HRQOL questionnaires. In subgroup exploratory analyses, a deleterious effect on FWB score was observed in patients with high level of aNK cells, but not statistically significant. A phase IIb–III study taking into account the pre-treatment level of aNK cells has been initiated to prospectively validate these clinical results and further analyze the evolution of HRQOL under chemo-immunotherapy with an acceptable statistical power (NCT01383148).

In conclusion, this study did not show significant differences in HRQOL over the course of the treatment between patients having received or not TG4010 in combination with chemotherapy and this despite the fact that the group of patients who received the vaccine presented a lower HRQOL at baseline. This supports the idea that TG4010 can be combined to first-line chemotherapy without deterioration in patients’ HRQOL. Understanding the relative effects of new treatment on HRQOL is important for effective decision making in this setting [12]. Future research should incorporate HRQOL as a treatment outcome for therapeutic cancer vaccines as well, particularly when the treatment is palliative.

## Supporting Information

S1 Authorization List of Ethics committees. (DOCX)

S1 Checklist CONSORT checklist. (DOC)

S1 Data Individual-level data. (ZIP)

S1 Fig Time to a five-point definitive deterioration in Health-related Quality of Life score or death for patients with a normal level of activated Natural Killer (aNK) cells: (A) Physical Well-Being dimension (PWB); (B) Social Well-Being dimension (SWB); (C) Emotional Well-Being dimension (EWB); (D) Functional Well-Being dimension (FWB); (E) Lung Cancer Subscale (LCS). Arm 1: combination therapy arm; Arm 2: control arm.
(TIF)

S2 Fig Time to a five-point definitive deterioration in Health-related Quality of Life score or death for patients with a high level of activated Natural Killer (aNK) cells: (A) Physical Well-Being dimension (PWB); (B) Social Well-Being dimension (SWB); (C) Emotional Well-Being dimension (EWB); (D) Functional Well-Being dimension (FWB); (E) Lung Cancer Subscale (LCS). Arm 1: combination therapy arm; Arm 2: control arm.
(TIF)

S1 Protocol Protocol of the study. (PDF)
