# Supplementary material for: Impact of TG4010 Vaccine on Health-Related Quality of Life in Advanced Non-Small-Cell Lung Cancer: Results of a Phase IIB Clinical Trial
Source: PLoS One. 2015 Jul 24;10(7):e0132568. doi: 10.1371/journal.pone.0132568 (PMC4514809; doi:10.1371/journal.pone.0132568)
Supplement: S1 Authorization — (DOCX) [file pone.0132568.s001.docx]

**LIST OF ETHICS COMMITTEES**

**FRANCE**: Comité Consultatif de Protection des Personnes dans la Recherche Biomédicale d’Alsace, 1, place de l’Hôpital, 67000 STRASBOURG cedex

| **Submitted documents** | **Submission n°** | **Date of submission** | **Date of approval** |
| --- | --- | --- | --- |
| Protocol Final version 08 MAY 2005* | Initial request | 19 MAY 2005 | 14 JUN 2005 |
| Protocol Final version 08 JUL 2005 | Additional request n°1 | 22 SEP 2005 | 11 OCT 2005 |

** Protocol final version 08 May 2005 has been submitted to French IEC and MoH but never applied, as the protocol has been revised during the MoH’s review. Nevertheless, IEC review is shorter than MoH review, then an IEC’s approval was obtained for this version of protocol.*

**GERMANY**

| **Investigator / Site** | **Central IEC** | **Name of the committee chair** |
| --- | --- | --- |
| All German sites | Site n° 306  Ethik-Kommission bei der Landesärztekammer  Baden-Württemberg Jahnstraße 40  **70597 Stuttgart** | Dr Petra Knupfer |

| **Site n°** | **Local IEC** |
| --- | --- |
| 304 | Medizinische Ethik-Kommission II  Medizinische Fakultät Mannheim  der Ruprecht-Karls-Universität  Heidelberg  Geschäftsstelle  Maybachstraße 14  **68169 Mannheim** |
| 305 | Ethikkommission der Medizinischen Fakultät Heidelberg  Alte Glockengießerei 11/1  **69115 Heidelberg** |
| 307 | Ethik-Kommission der  Ärztekammer Nordrhein – Westfalen  Tersteegerstr. 9  **40474 Düsseldorf** |
| 308 | Ethik-Kommission der Medizinischen Fakultät der Ludwigs-Maximilians Universität München  Marchioninistr.15  **81377 München** |
| 309 | Ärztekammer Schleswig-Holstein  Ethik-Kommission  Bismarckallee 8-12  **23795 Bad Segeberg** |
| 310 & 312 | Landesamt für Gesundheit und Soziales  Geschäftsstelle der Ethik-Kommission des Landes Berlin  Sächsische Straße 28  **10107 Berlin** |

| **Submitted documents** | **Date of submission** | **Date of approval** |
| --- | --- | --- |
| Amendments n°1, 2 and 3.  Protocol version A 14 FEB 2006 | 24 MAR 2006 | 19 JUN 2006 |

**POLAND**

| **Investigator / Site** | **IEC** | **Name of the committee chair** |
| --- | --- | --- |
| All Polish sites | KOMISJA BIOETYCZNA  Ethics Committee  PRZY OKRĘGOWEJ RADZIE LEKARSKIEJ  Attached to Okręgowa Rada Lekarska  WIELKOPOLSKIEJ IZBY LEKARSKIEJ  Regional Physician’s Council of Wielkopolska Physician’s Chamber  Ul. Nowowiejskiego 51  **61-734 Poznań** | Dr Maria de Mezer-Dambek |

| **Submitted documents** | **Date of submission** | **Date of approval** |
| --- | --- | --- |
| Final protocol 08 JUL 2005  Amendment n°1 21 OCT 2005 | 18 NOV 2005 | 30 NOV 2005 |

**HUNGARY**

| **Investigator / Site** | **Central IEC** | **Name of the committee chair** |
| --- | --- | --- |
| All Hungarian sites | EGÉSZSÉGÜGYI  TUDOMÁNYOS TANÁCS  Medical Research Council  KLINIKAI FARMAKOLOGIAI  ETIKAI BIZOTTSAGA  Ethics Committee for Clinical Pharmacology  Arany J.u.6-8  **1051 Budapest** | Prof. Dr. Fenyvesi Tamás  *and then*  Dr. Fürst Zsuzsanna |

| **Site n°** | **Local IEC** |
| --- | --- |
| 420 | Uzsoki utcai Hospital  Institutional Ethics Committee for Clinical Research  Uzsoki str. 29-41  **1145 Budapest** |
| 421 | Pest County Institute of Pulmonology  Local Ethics Committee  Munkácsy Mihály str. 70  **2045 Törökbálint** |
| 423 | Medical Scientific Board  Regional Ethics Committee for Clinical Research  Győr-Moson-Sopron-Komárom-Esztergom County  Vasvári Pál str. 2  **9024 Győr** |
| 424 | Fejér County St. George Hospital  Local Ethics Committee  Seregélyesi str. 3  **8000 Székesfehérvár** |

| **Submitted documents** | **Date of submission** | **Date of approval** |
| --- | --- | --- |
| Final protocol 08 JUL 2005  Amendment n°1 21 OCT 2005 | 28 DEC 2005 | 01 FEB 2006 |
