# Supplementary figures and images for: Impact of TG4010 Vaccine on Health-Related Quality of Life in Advanced Non-Small-Cell Lung Cancer: Results of a Phase IIB Clinical Trial
Source: PLoS One. 2015 Jul 24;10(7):e0132568. doi: 10.1371/journal.pone.0132568 (PMC4514809; doi:10.1371/journal.pone.0132568)

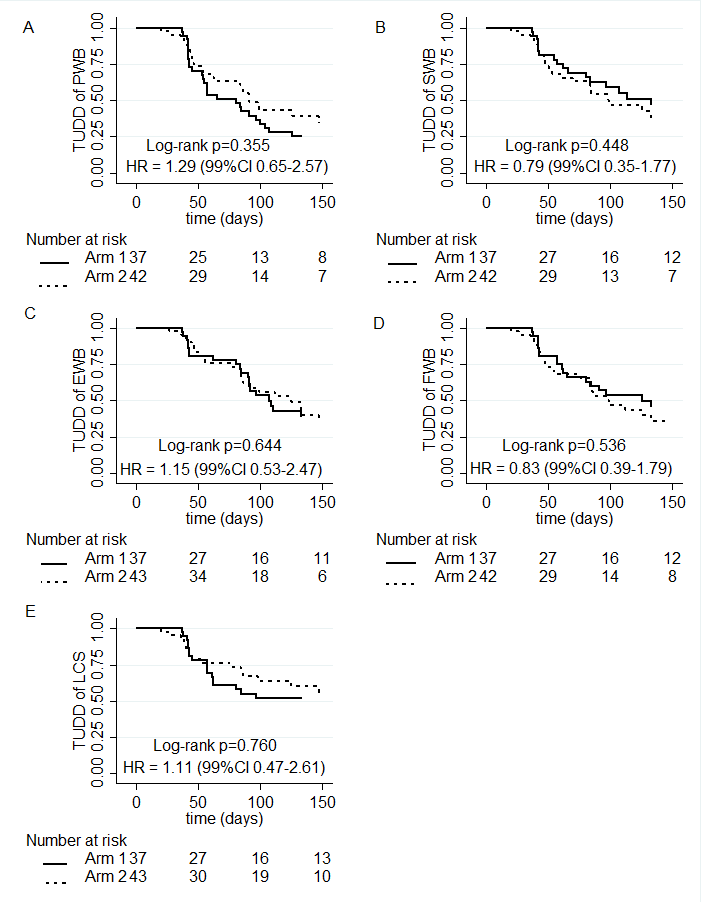

Supplement: S1 Fig — Time to a five-point definitive deterioration in Health-related Quality of Life score or death for patients with a normal level of activated Natural Killer (aNK) cells: (A) Physical Well-Being dimension (PWB); (B) Social Well-Being dimension (SWB); (C) Emotional Well-Being dimension (EWB); (D) Functional Well-Being dimension (FWB); (E) Lung Cancer Subscale (LCS). Arm 1: combination therapy arm; Arm 2: control arm. (TIF) [file pone.0132568.s004.tif]

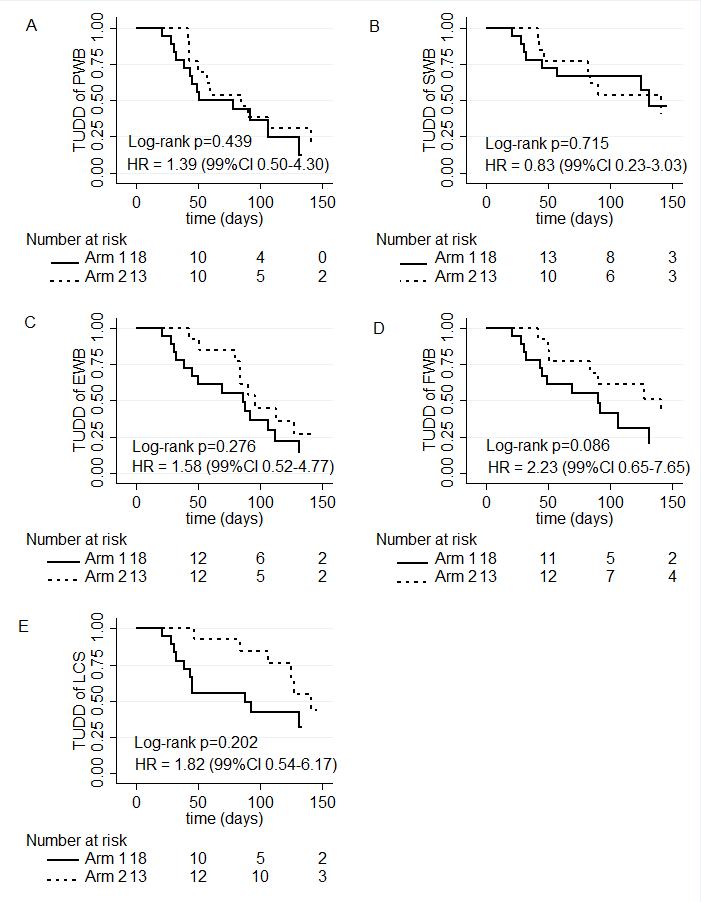

Supplement: S2 Fig — Time to a five-point definitive deterioration in Health-related Quality of Life score or death for patients with a high level of activated Natural Killer (aNK) cells: (A) Physical Well-Being dimension (PWB); (B) Social Well-Being dimension (SWB); (C) Emotional Well-Being dimension (EWB); (D) Functional Well-Being dimension (FWB); (E) Lung Cancer Subscale (LCS). Arm 1: combination therapy arm; Arm 2: control arm. (TIF) [file pone.0132568.s005.tif]
