# Supplementary material for: Impact of TG4010 Vaccine on Health-Related Quality of Life in Advanced Non-Small-Cell Lung Cancer: Results of a Phase IIB Clinical Trial
Source: PLoS One. 2015 Jul 24;10(7):e0132568. doi: 10.1371/journal.pone.0132568 (PMC4514809; doi:10.1371/journal.pone.0132568)
Supplement: S1 Protocol — (PDF) [file pone.0132568.s006.pdf]

Clinical Study Protocol

CONFIDENTIAL

A PHASE IIb MULTICENTRIC CONTROLLED STUDY EVALUATING THE THERAPEUTIC VACCINE TG4010 (MVA-MUC1-IL2) AS AN ADJUNCT TO STANDARD CHEMOTHERAPY IN ADVANCED NON SMALL CELL LUNG CANCER

PROTOCOL N° TG4010.09
Final Version 08 July 2005

Study phase: IIb

PRINCIPAL INVESTIGATOR: Prof. Elisabeth QUOIX, MD
Hôpital Lyautey
Service de Pneumologie
1, rue des Canonniers
67100 STRASBOURG, France
Phone: +33.(0)3.88.11.50.37
Fax: +33.(0)3.88.11.63.35

SPONSOR: **Transgene S.A.**
11, rue de Molsheim
67082 STRASBOURG Cedex, France

PERSON TO CONTACT: Dr Jean-Marc LIMACHER, MD
Clinical Project Manager
Medical and Regulatory Affairs Department
Transgene S.A.
Phone: + 33.(0)3.88.27.91.73
Fax: + 33.(0)3.88.27.91.41

The confidential information contained in this document is the property of Transgene. It is provided to you in confidence, for review by you, your staff, regulatory authorities, and members of ethics committees or institutional review boards. It is understood that this information will not be disclosed to any other third party, in any form, without prior authorization from Transgene, except to the extent necessary to obtain the written informed consent from the persons to whom the study drug may be administered.

INVESTIGATORS / STUDY ADMINISTRATIVE STRUCTURE

NAMES AND CONTACT DETAILS

Principal / Coordinating Investigator

Prof. Elisabeth QUOIX, MD
Hôpital Lyautey
Service de Pneumologie
STRASBOURG, France

Centers / Investigators

The complete investigator list is available in the investigator site file and in the trial master file

Transgene's Staff

Clinical Project Manager	Pharmacovigilance	Clinical Operations
Jean-Marc LIMACHER, MD<br>limacher@transgene.fr	Isabelle DIDILLON, PharmD<br>didillon@transgene.fr	Céline HALLUARD<br>halluard@transgene.fr<br>Gisèle LACOSTE<br>lacoste@transgene.fr
Medical and Regulatory Affairs<br>Transgene S.A.<br>11, rue de Molsheim<br>67082 STRASBOURG Cedex, France<br>Phone: + 33 (0)3.88.27.91.73<br>Fax: + 33 (0)3.88.27.91.41		

Statistician

Marc BUYSE
IDDI
430 avenue Louise
B14,
1050 BRUSSELS, Belgium
Phone: + 32 (0)2. 646.89.18
Fax: + 32 (0)2. 646.86.62

A complete list of details for the Clinical Research Organisation (CRO), the central laboratory/ies is available in the investigator site file and in Transgene files.

During the study, if applicable, the administrative structure will be updated in the investigator site file and in Transgene files.

DOCUMENT APPROVAL

<u>SPONSOR'S OFFICER(S)</u>

Patrick SQUIBAN, MD
Vice President,
Medical and Regulatory Affairs
Transgene S.A.

Signature Date

Jean-Marc LIMACHER, MD
Clinical Project Manager
Transgene S.A.

Signature Date

Marc BUYSE, ScD,
Statistician
IDDI

Signature Date

SYNOPSIS

**Sponsor:** Transgene S.A.<br>**Study Drug:** TG4010 (MVA-MUC1-IL2)	**Clinical Protocol:** TG4010.09

Study Title: A Phase IIb multicentric controlled study evaluating the therapeutic vaccine TG4010 (MVA-MUC1-IL2) as an adjunct to standard chemotherapy in advanced Non Small Cell Lung Cancer

Principal Investigator: Prof Elisabeth QUOIX (Strasbourg, France)

Investigational Centres (participating countries):

- France
- Belgium
- Poland

**Study Period:** Q4 2005 to Q4 2007	**Clinical Phase:** IIb

Objectives:

Primary Objective: to assess the efficacy of TG4010 combined to chemotherapy in comparison with chemotherapy alone in patients with advanced non small cell lung cancer. Efficacy will be assessed by progression free survival (PFS) at 6 months.

Secondary Objectives:

- To determine the response rate (RR), the time to progression (TTP) and the overall survival (OS)
- To confirm the safety of TG4010 administered subcutaneously in combination with chemotherapy in this patient population;
- To assess the immunological response;
- To explore the plasma proteome profile.

Methodology:

This is a randomized, open-label, multicenter study, testing TG4010 subcutaneous injections at the dose of 10^8 pfu in combination with chemotherapy versus chemotherapy alone.

The chemotherapy chosen for the study is cisplatin-gemcitabine for both experimental and control arms. Chemotherapy cycles will be initiated every 3 weeks and given for up to 6 cycles or progressive disease, whichever occurs first.

- In arm 1, patients will receive TG4010 once per week for 6 weeks then once every 3 weeks in combination with chemotherapy and then as monotherapy until documentation of progressive disease.
- In arm 2, patients will receive chemotherapy alone.

Tumor response will be evaluated every 6 weeks.

Number of Patients:

In total, this randomized phase IIb trial will enrol 140 patients to produce 2 groups of patients evaluable for progression free survival (PFS) at 6 months.

Diagnosis and Main Inclusion Criteria:

- Histologically confirmed stage IIIB “wet” (with pleural or pericardic effusion) or IV non-small cell carcinoma of the lung (adenocarcinoma, squamous cell carcinoma, or large cell carcinoma), positive for MUC1;
- No prior systemic treatment for advanced disease;
- With at least one measurable lesion evaluated by CT-scan according to WHO critera;
- Performance Status 0 or 1 on the ECOG scale;
- Minimum estimated life expectancy of 4 months.

Main Exclusion Criteria:

- History of any form of systemic therapy for non-small cell carcinoma of the lung except for (neo)adjuvant treatment;
- Concomitant brain metastasis;
- History of other malignancy in the past 5 years except for basal cell carcinoma and intra-epithelial cervical neoplasia.

Test Product, Dose, Mode of Administration:

- TG4010 is a viral suspension of a recombinant vaccinia vector (Modified Virus Ankara, a significantly attenuated strain of vaccinia virus), containing sequences coding for the human MUC1 antigen and human Interleukin-2 (IL2). In arm 1, patients will receive subcutaneous

**Sponsor:** Transgene S.A.<br>**Study Drug:** TG4010 (MVA-MUC1-IL2)	**Clinical Protocol:** TG4010.09

injections (SC) of TG4010 at the dose of 10^8 pfu.

Associated Therapy:

- Intravenous chemotherapy: Cisplatin: 75mg/m² (D1) + Gemcitabine 1250mg/m² (D1, D8), every 3 weeks, up to 6 cycles.
- The use of the following drugs will not be restricted during the course of the study: G-CSF, Erythropoietin, zetrons, steroids (short treatment only, chronic exposure to be excluded), biphosphonates, amifostine.

Duration of Treatment: End of treatment will be defined as disease progression.

Criteria for Evaluation:

1/ Efficacy:

- Primary endpoint: progression free survival at 6 months (PFS).
- Secondary endpoints: response rate (RR) according to WHO criteria, time to progression (TTP) and overall survival (OS).

2/ Safety: Adverse events and serious adverse events.

3/ Immunology: Cellular immune response assessed by tetramer and immunophenotype analysis.

4/ Translational Research: Proteome analyses by mass spectrometry.

Considered Statistical Methods

The trial will be conducted as two parallel single arm trials, with randomization and with PFS as primary end point. 140 patients should be included to obtain 67 evaluable patients in each arm.

The study is designed with the following assumptions (Fleming plan):

- H_0=30%, H_A=50%
- The risks α and β are set at 5%
- Treatment of 67 patients in the combination arm: if at least 27 patients (40%) are free of progression at 6 months the treatment will be declared effective.

The time to progression and overall survival will be calculated using the Kaplan-Meier method and compared using the log-rank test.

TABLE OF CONTENTS

INVESTIGATORS / STUDY ADMINISTRATIVE STRUCTURE **2**

DOCUMENT APPROVAL **3**

SYNOPSIS **4**

TABLE OF CONTENTS **6**

ABBREVIATIONS / DEFINITION OF TERMS **9**

1 INTRODUCTION **11**

1.1 BACKGROUND 11
1.2 STUDY DRUG RATIONALE 12
1.2.1 Specific tumor antigen MUC1 .... *12*
1.2.2 Interleukin 2 (IL2) *12*
1.2.3 The parental virus: Modified Virus of Ankara (MVA) *12*
1.3 PREVIOUS CLINICAL RESULTS 13
1.3.1 Phase I studies *13*
1.3.2 Phase II studies *14*
1.4 RATIONALE FOR CONDUCTING THE STUDY 16
1.5 POTENTIAL RISKS ASSOCIATED WITH TG4010 16
1.5.1 Risks associated with MVA *16*
1.5.2 Risks associated with TG4010 .... *16*
1.5.3 Viral dissemination of TG4010 .... *17*
1.6 RISKS ASSOCIATED WITH THE CHEMOTHERAPY 17

2 OBJECTIVES **18**

2.1 PRIMARY OBJECTIVE 18
2.2 SECONDARY OBJECTIVE(S) 18

3 STUDY DESIGN **18**

3.1 OVERALL STUDY DESIGN AND PLAN DESCRIPTION 18
3.1.1 Overall design and control methods *18*
3.1.2 Number of centers and patients *18*
3.1.3 Patient accrual and duration of study *19*
3.2 DISCUSSION OF STUDY DESIGN 19

4 STUDY POPULATION **20**

4.1 INCLUSION CRITERIA 20
4.2 EXCLUSION CRITERIA 20
4.3 CONCOMITANT DISEASES 21
4.4 CONCOMITANT THERAPY 21
4.4.1 Concomitant medications *21*
4.4.2 Therapy restrictions *21*

5 STUDY DRUG **22**

5.1 CHARACTERISTICS AND SUPPLY 22
5.2 PACKAGING AND LABELING 22
5.3 CONDITIONS OF STORAGE AND USE 23
5.4 PREPARATION FOR ADMINISTRATION 23

6 TREATMENT PLAN **24**

6.1 TREATMENT ADMINISTERED 24
6.1.1 Treatment regimens *24*
6.1.2 Administration of the study drug *25*
6.1.3 Duration of treatment and observation period *25*
6.2 METHOD OF ASSIGNING PATIENTS TO TREATMENT GROUPS 25
6.2.1 Recruitment and Randomization *25*

6.2.2 Stratification / Minimizations 26
6.2.3 Dose modifications 26
6.3 TREATMENT COMPLIANCE, DISPENSING AND ACCOUNTABILITY 26
6.4 PREMATURE WITHDRAWAL OF PATIENTS 26
6.4.1 Circumstances 26
6.4.2 Replacement policy 27

7 STUDY VISITS AND PROCEDURES 27

7.1 EVALUATIONS DESCRIPTION 27
7.2 BASELINE 28
7.3 WHILE ENROLLED ON THE PROTOCOL 29
7.3.1 First 6-week period 29
7.3.2 Subsequent 6-week periods 30
7.4 END OF STUDY VISIT 31
7.5 POST-STUDY FOLLOW-UP 31
7.6 HANDLING OF BIOLOGICAL SAMPLES 31

8 ASSESSMENT OF EFFICACY 31

8.1 PRIMARY VARIABLE 31
8.2 SECONDARY VARIABLE(S) 32
8.3 APPROPRIATENESS OF MEASUREMENTS 32
8.4 REVIEW OF THE PATHOLOGICAL SAMPLES 32

9 ASSESSMENT OF SAFETY 32

9.1 DEFINITIONS 32
9.2 INTENSITY, RELATIONSHIP AND OUTCOME EVALUATION 34
9.3 PRE-DRUG EVENT MANAGEMENT 35
9.4 ADVERSE EVENT MANAGEMENT 36
9.5 SERIOUS ADVERSE EVENT MANAGEMENT 37
9.6 LABORATORY VALUES, VITAL SIGNS, PHYSICAL FINDINGS AND OTHER SAFETY DATA 38

10 STATISTICAL METHODS PLANNED AND SAMPLES SIZE 38

10.1 DETERMINATION OF SAMPLE SIZE 38
10.2 CONTINUATION OF TRIAL IN PHASE III 39
10.3 STUDY ENDPOINTS 39
10.4 STATISTICAL AND ANALYTICAL PLAN 40
10.5 DISPOSITION AND REPLACEMENT OF PATIENTS 40
10.6 PROTOCOL DEVIATIONS 40
10.7 DATA SETS ANALYZED 41

11 CHANGES IN THE CONDUCT OF THE STUDY 41

11.1 PROTOCOL AMENDMENTS 41
11.2 PREMATURE STUDY TERMINATION 41

12 ETHICAL CONSIDERATIONS 41

12.1 INDEPENDENT ETHICS COMMITTEE 41
12.2 INFORMED CONSENT 42
12.3 CONFIDENTIALITY OF PATIENT DATA 43

13 REGULATORY CONSIDERATIONS 43

13.1 REGULATORY CONSIDERATIONS 43
13.2 REGULATORY APPROVAL / AUTHORIZATION 43
13.3 INVESTIGATORS OBLIGATIONS 43
13.4 INSURANCE 44

14 QUALITY CONTROL AND QUALITY ASSURANCE 44

14.1 SOURCE DATA AND DOCUMENTS 44
14.2 PERIODIC MONITORING 44
14.3 AUDIT AND INSPECTION 44

15 DATA HANDLING AND RECORD KEEPING ... 45
15.1 INVESTIGATORS INFORMATION ... 45
15.2 CASE REPORT FORMS ... 45
15.3 CHANGES TO CASE REPORT FORM DATA ... 45
15.4 PROVISION OF ADDITIONAL INFORMATION ... 45
16 REPORTING AND PUBLICATION ... 46
16.1 CLINICAL STUDY REPORT ... 46
16.2 CONFIDENTIALITY OF STUDY DATA ... 46
16.3 PUBLICATION POLICY ... 46
17 ARCHIVING ... 46
17.1 INVESTIGATOR SITE FILE ... 46
17.2 TRIAL MASTER FILE ... 47
18 REFERENCES ... 47
19 APPENDICES ... 49

ABBREVIATIONS / DEFINITION OF TERMS

ABBREVIATIONS	MEANING OF ABBREVIATIONS IN DOCUMENT
AE	Adverse Event
ALT	Alanine amino-transferase (= SGOT)
AST	Aspartate amino-transferase (= SGPT)
CEF	Chick embryo fibroblasts
CR	Complete Response
CRF	Case Report Form
CRO	Contract Research Organization
CRP	C-Reactive Protein
CTCAE	Common Terminology Criteria for Adverse Events
CTL	Cytotoxic T Lymphocyte
ECOG	Eastern Cooperative Oncology Group
FACT-L	Functional Assessment of Cancer Therapy-Lung
γGT	Gamma Glutamyl Transferase
GCP	Good Clinical Practice
G-CSF	Granulocyte Colony Stimulating Factor
GMO	Genetically Modified Organism
HBs	Hepatitis B Virus Antigen s
HCV	Hepatitis C Virus
HIV	Human Immunodeficiency Virus
ICH	International Conference on Harmonization
ID	Intra-dermal
IEC	Independent Ethics Committee
IL2	Interleukin-2
IM	Intramuscular
LAK	Lymphokine activated killer
LDH	Lactate deshydrogenase
MUC1	Mucine 1
MVA	Modified Virus of Ankara
MW	Molecular Weight
NK	Natural Killer
NSCLC	Non Small Cell Lung Cancer
OS	Overall Survival
PCR	Polymerase Chain Reaction
PD	Progressive Disease
PDE	Pre-Drug Event
PFS	Progression Free Survival
pfu	plaque forming unit
PR	Partial Response
PS	Performance Status
PSA	Prostate Specific Antigen
PTT	Prothrombin time
RBC	Red Blood Cells
RR	Response rate
SAE	Serious Adverse Event
SC	Subcutaneous
TAA	Tumour Associated Antigen
TPO	ThyroPerOxydase
TTP	Time To Progression
WBC	White Blood Cells
WHO	World Health Organization

ABBREVIATIONS / DEFINITION OF TERMS (Cont.)

DEFINITIONS

Completed patient	A patient who has completed all procedures up to the "end of study visit" as planned by the study protocol.
Consented patient	A patient who has signed the informed consent form.
Evaluable patient	A patient evaluable for disease progression during the 6 first months following the date of randomization.
Lost of follow-up patient	An included patient for whom no further news is obtained by the investigator before the "end of study visit" has been performed. The date of lost of follow-up is the date the investigator received the last news from the patient i.e. during a visit or a telephone contact or any written means.
Not included patient	A not included patient is a consented patient not randomized in the study for any reason.
Ongoing patient	A patient included, presently treated according to the protocol requirements.
Randomized/Included patient	A patient that signed the ICF, satisfied all inclusion and exclusion criteria after the baseline visit procedures and who was assigned a study arm and study number.<br>A randomized patient is a patient included in the study.
Screened patient	A patient identified during the screening process achieved by the investigator as a candidate to the study and invited to sign the informed consent form.
Treated patient	A patient having received at least the first study drug administration as planned by the protocol.
Withdrawn patient<br>(= drop-out patient)	An included patient who leaves the study between the inclusion date and the end of study visit whatever the reason.
End of study	The date of the last visit of the last patient undergoing the study.
Protocol deviation	All non adherences to following protocol requirements: study inclusion or exclusion criteria, conduct of the study, patient management or patient assessment.

A PHASE IIb MULTICENTRIC CONTROLLED STUDY EVALUATING THE THERAPEUTIC VACCINE TG4010 (MVA-MUC1-IL2) AS AN ADJUNCT TO STANDARD CHEMOTHERAPY IN ADVANCED NON SMALL CELL LUNG CANCER

1 INTRODUCTION

1.1 Background

Lung cancer is one of the most common malignancies worldwide and the leading cause of cancer-related deaths in both men and women with respectively 31% and 27% of cancer death {Jemal A., 2005}. According to the World Health Organization, there are more than 1.2 million cases of lung and bronchial cancer diagnosed each year worldwide, causing approximately 1.1 million deaths annually {Parkin D.M., 2004}. Non–small-cell lung cancer (NSCLC) accounts for approximately 80% of cases. Current treatments for lung cancer include surgery, chemotherapy, radiation and targeted molecular therapy but only a third of patients present with resectable disease at diagnosis. With best supportive care only, less than 10% of patients survive for 1 year, and median survival is 5 to 6 months {Ginsberg R.J., 2001}. This poor prognosis in patients with advanced disease is improved by palliative platinum based chemotherapies {NCLC, 1995, Oct 7}.

In the past decade, platinum compounds have been combined with newer chemotherapy agents, such as vinorelbine, gemcitabine, paclitaxel, docetaxel and irinotecan. These combinations produce higher response rates and longer survival times and thus have become the gold standard for first-line treatment of advanced NSCLC. Despite their contributions, a therapeutic plateau has been reached, with response rates seldom exceeding 30-40% and 1-year survival rates stable between 30 and 40% {Langer C.J., 2004}.

The combination of three drugs including cisplatin does not improve the results of a two-drug chemotherapy in advanced disease {Delbaldo C., 2004}.

As several molecular targets for NSCLC have been identified, a number of new biologic agents have been developed and introduced in clinical trials.

The anti-epidermal growth factor receptor (EGFR) group includes gefinitib and erlotinib that showed antitumor activity in the second-line setting but failed to demonstrate a benefit in the first-line setting when added to standard chemotherapy regimens {Langer C.J., 2004}. Cetuximab, a monoclonal antibody that inhibits EGFR ligand binding, has demonstrated a synergy with chemotherapeutic agents in patients with colon cancer and head and neck cancer and is under evaluation in patients with NSCLC {Govindan R., 2004}.

Bevacizumab is a monoclonal antibody directed against vascular endothelial growth factor (VEGF). In a previous study in NSCLC, the addition of bevacizumab to standard carboplatin/paclitaxel chemotherapy increased the time to progression with a non significant increase in response rate. It is actually investigated in a randomized study in comparison with standard chemotherapy alone {Johnson D.H., 2004}.

Pemetrexed is a multitargeted antifolate agent with antitumor activity. In a randomized phase III trial comparing pemetrexed with docetaxel as second-line therapy in advanced NSCLC it

has shown similar activity with less toxicity {Hanna N., 2004, May 1}. Additional trials are planned.
The medical need in NSCLC is enormous and new approached are necessary to change significantly the outcome of this disease. Immunotherapy by modifying the host/tumor relations may achieve such a result.

1.2 Study drug rationale

An immunotherapy in NSCLC need to mount or amplify an immune response against one or several epitopes present in the tumor. MUC1 (Mucin 1) a tumor-associated antigen (TAA) is such a potential target for immunotherapy of lung cancer.
TG4010 is a viral suspension of a recombinant vaccinia vector containing the sequences coding for the human MUC1 antigen and for human Interleukin-2 (IL2). The product TG4010 has been developed for use as an immunotherapy in cancer patients whose tumors express the MUC1 antigen.

1.2.1 Specific tumor antigen MUC1

The human MUC1 protein, a highly glycosylated mucin (MW > 200 kD), is a constituent of the glycocalyx normally found at the apical surface of mucin-secreting epithelial cells in many types of tissues, including the breast, prostate, lungs, pancreas, stomach, ovaries, fallopian tubes, intestine, kidney and much others {Peat N., 1992}, {Fujita K., 1999}{Gansbacher B.K., 1990}. Tumors originating from these secretory epithelial cells often over-express MUC1 {Hareuveni M., 1990}; {Ho S.B., 1993}; {Layton G.T., 1990}. The most important characteristics of MUC1 protein in tumors compared to normal tissues is the much reduced glycosylation revealing new peptide and carbohydrate epitopes {Burchell J., 1987}; {Devine P., 1990}. This immunological difference between MUC1 in normal cells and in tumors makes it a target for immunotherapy. MUC1 seems also to be positively selected during the tumor progression and for this reason therapeutic vaccination against MUC1 may be efficient even in advanced disease.

1.2.2 Interleukin 2 (IL2)

TG4010 contains also a cDNA sequence which codes for human IL2. This cytokine has important functions in the immune response, as IL2 has been shown to be an essential factor in cell-mediated and humoral immune responses {Kaplan G., 1992}.
Additionally, IL2 activates the cytotoxic function of LAK and NK cells and memory CTLs {Bubenik J., 1993}, {Foa R., 1992}, {Gansbacher B.K., 1990}.
Local administration via a recombinant vaccinia virus produces very low levels in the circulation greatly reducing the risk of adverse side effects associated with systemic administration of IL2 {Konrad M.W., 1990}.

1.2.3 The parental virus: Modified Virus of Ankara (MVA)

The carrier chosen for TG4010 is MVA which belongs to the poxvirus family. Poxviruses form a complex family of DNA viruses which infect both vertebrates and invertebrates. The best-known member of the poxvirus family is the smallpox virus {Behbehani A.M., 1983}. Immunization with live vaccinia virus led to smallpox eradication. MVA is such a live vaccine that has been designed for vaccination against smallpox.

MVA is a highly attenuated virus strain derived from the vaccinia virus strain Ankara by over 570 passages in chick embryo fibroblast (CEF) cells. During these passages on CEF cells, MVA lost approximately 15% of the viral DNA from the wild type strain. As a consequence, MVA lost the ability to productively infect several types of mammalian cells {Mayr A., 1975}, {Mayr A., 1978}, {Carroll M.W., 1997}. This virus presents a severely restricted host range. It does not replicate in primary human fibroblasts. Early *in vivo* testing of MVA revealed the absence of toxicity, both in normal and immunosuppressed animals.

MVA has been developed in Germany, in the 1970s, to specially vaccinate high risk subjects against smallpox. More than 150,000 people, including high risk patients with nervous system disorder, allergy or skin disease, chronic disease and young children, have been vaccinated with MVA, without safety concerns (mild redness at the injection site and a low percentage of subjects with fever greater than 38°C and/or general malaise) {Mahnel H., 1994}, {Stickl H., 1974}. Serious complications, such as encephalitis and disseminated infection, observed when live Vaccinia Virus was injected in previous smallpox vaccination campaigns, were not observed with MVA. Vaccination with MVA was performed according to several schedules of administration: either MVA used for primo-vaccination (ID, SC or IM) following by boost injection with Elstree (WHO reference at that time) or MVA injected IM for both primo-vaccination and boost. As observed during these vaccination campaigns, MVA is an efficient immunogen in spite of its attenuation {Stickl H., 1974}, {Mayr A., 1978}.

In addition, this deletion of viral genetic information provided additional space for the insertion of one or more transgenes {Sutter G., 1992}, {Sutter G., 1994}. Moreover, recombinant MVA vectors are able to produce high levels of foreign proteins in human cells {Sutter G., 1992}.

Further information about study drug is available in the Investigator Brochure.

1.3 Previous clinical results

1.3.1 Phase I studies

Two phase I dose escalation bridging studies have been completed with TG4010. Studies were performed in Basel (Kantonsspital) and Los Angeles (UCLA), in patients with advanced cancer positive for MUC1. Thirteen patients have entered these studies.
TG4010 was administered IM to sequential cohorts of three patients receiving the following dosing schedules: $5x10^6$ pfu every three weeks, $5x10^6$ pfu every weeks, $5x10^7$pfu every three weeks, and 10^8 pfu weekly for the third cohort. At every dose level the tolerance was good and no viral dissemination was observed. The dose 10^8 pfu was not the maximal tolerated dose but the maximal feasible dose for technical reasons (volume and concentration). This dose was retained for the following phase II studies. Four patients experienced disease stabilization. Ten patients were evaluable for the cellular immune response: 6 of them showed such an immune response against MUC1.

1.3.2 Phase II studies

TG4010.03: Randomized multicenter phase II study evaluating two dosing schedules of TG4010 (MVA-MUC1-IL2) in patients with adenocarcinoma of the prostate.

Patient population:
Men previously locally treated for their prostate cancer (surgery, radiotherapy or both) presenting with a biochemical failure (PSA$\geq$2ng/ml, PSA-doubling time $\leq$10months) without evidence of metastases.
Intervention:
TG4010 given SC as a monotherapy.
Endpoint:
PSA kinetics.
Dosing:
10^8pfu,
Arm 1: weekly for 6 injections then every 3 weeks,
Arm 2: every 3 weeks.
Treatment duration:
Until progression or up to 9 months.
Status:
Forty patients included, 6 active, enrolment closed.
Main findings (interim results March 05):
The majority of the patients (63%) increase their PSA-doubling time (proof of biological activity).
There is a trend in favour of the weekly schedule.

TG4010.04: Randomized, multicenter, phase II study evaluating two doses of TG4010 (MVA-MUC1-IL2) in patients with metastatic breast cancer

Patient population:
Women with a progressive metastatic MUC1 positive breast cancer after at least one line of chemotherapy.
Intervention:
TG4010 given SC as a monotherapy.
Endpoint:
Response rate.
Dosing:
Weekly for 6 injections, then every 3 weeks,
Arm 1: 10^8 pfu,
Arm 2: 10^6 pfu.
Treatment duration:
Until progression or up to 12 months.
Status:
Forty two patients included, study completed.
Main findings:
No objective response but 13/42 (31%) of the patients were stable at 12 weeks. A decrease of the serum tumor marker CA15.3 has been observed in 9 patients.

TG4010.05: Randomized Multicenter Phase II Study Evaluating The Clinical Efficacy of TG4010 (MVA-MUC1-IL2) in Association With Chemotherapy in Patients With Non Small Cell Lung Cancer (NSCLC).

Patient population:
Patients with a stage IIIB-IV MUC1 positive NSCLC, first line.
Intervention:
TG4010 given SC as a monotherapy and in combination with chemotherapy (cisplatin 100mg/m² D1, vinorelbine 25mg/m² D1/D8).
Endpoint:
Response rate.
Dosing:
10^8 pfu, weekly for 6 injections then every 3 weeks until progression,
Arm 1: immediate combination with chemotherapy,
Arm 2: monotherapy with TG4010 until progression, then in combination with chemotherapy.
Treatment duration:
Until progression.
Status:
Sixty five patients included, 1 active, enrolment closed.
Main findings (interim results April 05):
Arm 1: 13/35 (37%) patients with a partial response validated through a central reading process. Time To Progression (TTP) = 6.4 months [4.1-6.7], Overall Survival (OS) = 13 months [9.6-15]. One year survival: 53%.
Arm 2: 2/18 patients stable more than 6 months with TG4010 alone. 2/14 with a partial response in subsequent combination therapy. TTP = 7.2 months [4.3-7.6], OS = 14.5 months [8.7-16.9] One year survival: 60%.
The cellular immune response against MUC1 for the whole study population was assessed in 31 patients. (ELISpot and T-cell proliferation at baseline, D43 and D64)
An immune response against MUC1 was detected in:
7/7 (100%) patients with PR,
9/13 (70%) patients with SD,
5/11 (45%) patients with PD.
The overall survival of the patients with a cellular immune response against MUC1 is significantly better as the overall survival from the patients without such a response. ($p=0.0035$)

TG4010.06: Phase II study evaluating the clinical efficacy of TG4010 (MVA-MUC1-IL2) in patients with progressive metastatic Renal Cell Carcinoma (RCC).

Patient population:
Patients with a metastatic MUC1 positive RCC.
Intervention:
TG4010 given SC as a monotherapy until progression then in combination with Interleukine 2 and Interferon α.
Endpoint:
Response rate.
Dosing:
10^8 pfu, weekly for 6 injections then every 3 weeks.

Treatment duration:
Until progression.
Status:
Thirty seven patients included, 3 active, enrolment closed.
Main findings (interim results March 05):
Four patients stable more than 6 months with TG4010 alone, two patients stable more than a year with the combination of TG4010 and cytokines. Median overall survival not reached at 13 months.

1.4 Rationale for conducting the study

The proof of biological activity of TG4010 has been obtained with the prostate cancer study TG4010.03.
In the lung cancer study TG4010.05 the endpoint has been over-passed.
This leads Transgene to consider the phase II program as positive and the product TG4010 worth to be further developed.
Concerning the safety of the product, no Serious Adverse Event related to TG4010 has been observed during the phase II program, minor to moderate injection site reactions being the most frequent adverse events.
For these reasons Transgene has decided to pursue the clinical development of TG4010 through a randomized study in NSCLC comparing the results of the first-line chemotherapy with and without TG4010.

1.5 Potential risks associated with TG4010

1.5.1 Risks associated with MVA

§ Immunization with MVA
For patients who have been immunized previously against smallpox with a vaccinia virus preparation (i.e. Elstree or Wyeth), the use of a cancer vaccine based on the MVA strain may pose even less of an issue, since the previous vaccination provides a further element of safety. For those patients who have not been vaccinated against smallpox the use of this MVA construct should be at least as safe as smallpox vaccination, particularly with the enhanced safety profile of MVA compared with other strains of vaccinia virus.

§ Possible adverse effects related to MVA
According to a field study carried out on over 7 000 primary vaccinees receiving ID the MVA strain (vaccinees included children under and over the age of three years), the only adverse events observed were: local reaction (redness) which demonstrated that patients responded to the immunization, fever (2% of vaccinees), ‘flu-like’ symptoms (4% of vaccinees). Contrary to what had been observed with other vaccinia strains, less adverse reactions were reported during the vaccination campaign with MVA (for more details, see Investigator Brochure).

1.5.2 Risks associated with TG4010

Potential autoimmune toxicity from TG4010 related to cross reactivity with natural MUC1 protein cannot be ruled out. However, no evidence of autoimmune phenomena has been reported in MUC1 transgenic animals following the administration of TG4010. Only one patient treated with the earlier generation product TG1031 (Vaccinia Virus-MUC1-IL2), and

with family history of thyroiditis, reported an increase of anti-TPO antibodies level, associated with occurrence of anti-DNA and anti-nuclear antibodies, mild fatigue and biological signs of hypothyroidism. This patient presented partial response to treatment (regression of hepatic metastases from breast cancer). Treatment was maintained for one year and stopped, after which patient could undergo surgery for her stabilized hepatic metastases. Signs of auto-immunity resolved spontaneously before treatment with TG1031 was interrupted.

IL2 protein, administered systemically, has been associated with a variety of toxicities. These toxicities have severely limited the therapeutic utility of systemic IL2.
IL2 protein expressed by TG4010 from the human IL2 coding sequence appears to be localized to the site of injection and expressed at a low level, and there has been no evidence of systemic IL2 toxicity associated with this product during toxicology testing.

In phase II studies, TG4010 is well tolerated and the main reactions reported are local injection site reactions, fatigue, anemia, nausea and vomiting.
Among the serious adverse events reported none of them was considered by investigators as related to TG4010 administrations.
Further information about adverse events is available in the Investigator Brochure.

1.5.3 Viral dissemination of TG4010

The thirteen patients treated with TG4010 in phase I were monitored by PCR viral dissemination in blood and urines samples {Schmidt I., 2000} {Schmidt I., 2001}. The limit of detection was around 90 pfu/ml in blood samples and around 400 pfu/ml in urine samples.
No presence of the viral genome was detected by PCR in the blood or urine samples.
In study TG4010.05, virology evaluation in blood and in urine was performed on days 1 and 22. The complete results of the PCR assays on 41 patients are negative; no viral dissemination has been observed.

1.6 Risks associated with the chemotherapy

The most common side effects of cytotoxic chemotherapy are:
- Myelotoxicity: leucopenia, neutropenia, anemia, thrombopenia;
- Nausea and vomiting, diarrhea;
- Peripheral neuropathy (cisplatin);
- Flu-like syndrome;
- Alopecia (cisplatin);
- Renal toxicity (cisplatin);
- Transient increase of transaminases (gemcitabine);
- Dyspnea (gemcitabine);
- Hematuria, proteinuria (gemcitabine);
- Ototoxicity (cisplatin);
- Allergy.

Other side effects are listed in the manufacturer’s Summery of Product Characteristics.

2 OBJECTIVES

2.1 Primary objective

The primary objective of this study is to assess the efficacy of TG4010 combined to chemotherapy in comparison with chemotherapy alone in patients with advanced non small cell lung cancer. Efficacy will be assessed by progression free survival (PFS) at 6 months.

2.2 Secondary objective(s)

Secondary objectives include:
- Determination of response rate (RR), time to progression (TTP) and overall survival (OS),
- Safety assessment of TG4010 administered subcutaneously together with chemotherapy in this patient population,
- Monitoring of immunological response,
- Exploration of plasma proteomics changes between patients and during therapy.

3 STUDY DESIGN

3.1 Overall study design and plan description

3.1.1 Overall design and control methods

This is a phase IIb randomized controlled, open-label and multicenter study, testing TG4010 subcutaneous injections at the dose of 10^8 pfu in combination with chemotherapy treatment versus chemotherapy alone. The chemotherapy associates cisplatin and gemcitabine. Chemotherapy cycles will be initiated every 3 weeks and given up to 6 cycles or progressive disease, whichever occurs first.
- In arm 1, patients will receive TG4010 once per week for 6 weeks then once every 3 weeks in combination with chemotherapy and then as monotherapy until documentation of progressive disease.
- In arm 2, patients will receive chemotherapy alone.

The chemotherapy is:

Cisplatin: 75mg/m² at day 1,
Gemcitabine: 1250mg/m² at days 1 and 8,
New cycle at day 22.

Tumor response will be evaluated every 6 weeks by CT-scan and results will be available before starting an additional treatment period of 6 weeks. The tumor response taken into account will be for each patient the best overall response obtained during the study.

3.1.2 Number of centers and patients

The study is planned to include a minimum of 140 patients in at least 15 centers in order to have 67 evaluable patients in each arms. Additional centers may be open in order to improve the recruitment.

3.1.3 Patient accrual and duration of study

This study is expected to start in Q4/2005 with recruitment to be completed by Q2/2007. The study is expected to be completed by Q4/2007.

It is understood that these accrual rates are based on reasonable planning expectations. The actual accrual rates should be compared to the expected rates on an ongoing basis. If problems with recruitment are encountered this should be discussed with Transgene as early as possible in order to institute measures to meet the above timelines.

3.2 Discussion of study design

Results observed in NSCLC with TG4010 in combination with chemotherapy are interesting and justify to evaluate the potential gain of adding TG4010 to chemotherapy through a randomized study. For this reason, this phase IIb study will evaluate the combination of TG4010 and chemotherapy with a control arm using the same chemotherapy given alone. This comparative approach starts with the present medium sized randomized study (Phase IIb) and may continue in phase III upon satisfaction of the endpoint or upon demonstration of a meaningful benefit for the experimental arm.

There is today no evident rationale for a chemotherapy specific effect of TG4010. In order to support this hypothesis a platinum based doublet different from the one tested in phase II and close to the European standard is used in the phase IIb study.

The dose of TG4010 used in the study is the dose determined by the phase I and used in the previous phase II. The schedule of vaccination is the one used in the previous phase II in the same indication. Based on the observations from the phase II of TG4010 in prostate cancer this weekly schedule of administration for the first six weeks is considered more efficient as an immediate three weekly vaccination.

The study is open to patients newly diagnosed with stage IV or IIIB with effusion NSCLC, and with PS 0 or 1, because a platinum based doublet with palliative intention is the standard of care in this situation. Their disease is to be positive in immuno-histo-chemistry for the presence of the vaccination target MUC1.

The endpoint of the study is based on Progression Free Survival (PFS) at 6 months. PFS has been chosen for three reasons:

- It reflects disease evolution even in the absence of an objective response.
- It can be assessed earlier as an effect on survival.
- It is not biased by subsequent lines of treatment

Six months is a time sufficient to allow the onset and the effect of a cellular immune response. The secondary parameters (RR, TTP, and OS) measured in each of both treatment arms will be compared also to completely assess the potential gain of adding TG4010 to the chemotherapy.

The WHO system of evaluation has been retained for two reasons:

- As many lesions in NSCLC are standing against pleura or mediastinum, these lesions may decrease in thickness under treatment while conserving the same greatest dimension (response may be underscored with RECIST).

- The data collected by the WHO system allows also a conversion of the response according to RECIST, the contrary being not possible.

A placebo of the vaccine is not possible because 80% of the patients treated with TG4010 present with minor to moderate injection site reactions.

The monitoring of the cellular immune response is aimed to prove the concept of vaccination against MUC1 while correlating this immune response with the result of the treatment.
Proteomic analysis by mass spectrometry is a technology allowing the identification within the numerous proteins of the plasma those predictive of the response to treatment or correlated with.

4 STUDY POPULATION

4.1 Inclusion criteria

Patients must satisfy all of the following criteria for entry into the protocol:

- Male or female patients, age ≥ 18 years old;
- Histologically confirmed non-small cell carcinoma of the lung (adenocarcinoma, squamous cell carcinoma, or large cell carcinoma);
- Histological documentation of MUC1 antigen expression on the primary tumor or on metastasis, as defined by a positive staining by immuno-histo-chemistry in at least 25% of the tumor cells in the conditions described in the technical documentation of the monoclonal antibody;
- Patients will have stage IIIB "wet" (with pleural or pericardic effusion) or IV disease, with no prior systemic therapy for advanced disease except for adjuvant treatment. Prior surgery or radiation therapy aimed at local palliation or attempted local disease control is permitted;
- At least one measurable lesion by Computed Tomography (CT-scanner) according to WHO criteria (lesion accurately measured in two dimensions with longest diameter equal or greater to 10 mm with spiral CT scan);
- Adequate hematological, hepatic, and renal function:
  - Hemoglobin ≥ 10.0 g/dL; WBC ≥ $3.0x10^9$/L including neutrophils ≥ $1.5x10^9$/L and total lymphocytes count ≥ $0.5x10^9$/L; platelets count ≥ $100x10^9$/L;
  - Bilirubin ≤ 2x the upper limit of normal and serum transaminases ≤ 3x the upper limit of normal;
  - Creatinine ≤ 2x the upper limit of normal;
- Performance status 0 or 1 on the ECOG scale (Appendix 2);
- Minimum estimated life expectancy of 4 months;
- Written informed consent from patient.

4.2 Exclusion criteria

Patients will be excluded from the study for any of the following reasons:

- Concomitant brain metastases. If previous brain metastases were treated, the absence of evolution is to be demonstrated by the MRI or scanner performed at baseline;

- Prior history of other malignancy except basal cell carcinoma and intra-epithelial cervical cancer or other cancer with complete response since at least 5 years;
- History of any form of systemic therapy for advanced non-small cell cancer of the lung except for (neo)adjuvant treatment;
- Previous (within 4 weeks prior to day 1) or concomitant long term treatment with systemic steroids, immunosuppressive / immunomodulating drugs (e.g. Cyclosporine, corticoïds);
- Positive serology for HIV or HCV; positive antigens for hepatitis B;
- Serious concomitant medical disorder;
- Major surgery within 4 weeks prior to day 1;
- Patient with an organ allograft;
- Allergy to eggs;
- Participation in another experimental protocol during the study period, or within 4 weeks prior to day 1;
- Pregnancy at the entry or women who are breast feeding;
- Patient without adequate protection against pregnancy during the conduct of the study and for 3 months after the last injection of TG4010 and/or chemotherapy;
- History of substance abuse;
- Patient unable or unwilling to comply with the protocol requirements.

4.3 Concomitant diseases

Concomitant diseases are diseases present at the baseline visit and not listed in the exclusion criteria (e.g. hypertension, diabetes). They should be under control at baseline.

Concomitant diseases will be reported in the case report form (CRF) with their treatments. Whenever possible, the treatments for concomitant diseases should not change during the course of the study. If this occurs, changes should be reported in the CRF.

4.4 Concomitant therapy

4.4.1 Concomitant medications

Pre-study medications are medications taken by the patient during the month before baseline and stopped before baseline.
Concomitant medications are treatments taken by the patient during the study and not listed in the exclusion criteria. They are reported in the CRF with their indications, start and stop dates.

4.4.2 Therapy restrictions

Any systemic therapy for advanced NSCLC is forbidden before and during study participation. However, (neo)adjuvant treatments are permitted.
Radiotherapy for pain release (e.g: bone metastases) is permitted. If target lesions are in the field of radiation they are no more evaluable for the tumor response. Of course, when necessary, effusions will be drained.
These supportive interventions will also be reported in the CRF.
Previous (within 4 weeks prior to Day 1) or concomitant long term treatment with systemic steroids, immunosuppressive / immunomodulating drugs (e.g. Cyclosporine, corticoïds) are not allowed.

However, systemic corticoids may be prescribed in prevention or treatment of nauseas and vomiting induced by the chemotherapy or in case of acute symptoms (asthma, inflammatory pain as arthritis…). This treatment should not exceed 3 days.
Treatment by G-CSF, EPO, biphosphonates, zetrons, amifostine are permitted during the study.

5 STUDY DRUG

5.1 Characteristics and supply

The study drug TG4010 is a viral suspension of a recombinant vaccinia vector containing sequences coding for the human MUC1 antigen and human IL2 (for further information see section 1.2 and the Investigator Brochure).

The study drug TG4010 will be supplied along with a Technical Sheet detailing its characteristics.
It will be produced and provided free of charge by Transgene.

5.2 Packaging and labeling

The study drug TG4010 is supplied in individual glass ampoules. Each ampoule is intended for single use (i.e. one injection to one patient) and is shipped in a secondary plastic container.

The primary label on the ampoules is in English. Secondary container labeling is in country specific language where the study is to be performed. Secondary containers are packed in a "Safepack".

The following is an example of the primary and secondary label to be used:

Primary labeling:

Product code: **TG4010**
Lot #: ZXXX
Ampoule #: XXX
Store at or below: -70°C
Reassessment date: XXXX
For clinical research only
TRANSGENE-FRANCE

Secondary labeling:

Lot: ZXXX	**Study: TG4010.09**<br>**Site: XXX**
	**Product code: TG4010**

Storage: temperature at or below –70°C
Retest date: see certificate of shelf-life extension
Volume / ampoule: xxx ml **Concentration:** xxx pfu/ml
Route of administration: subcutaneous
Conditions of use: see technical sheet
For clinical trial use only

TRANSGENE
11, rue de Molsheim - 67082 STRASBOURG Cedex - FRANCE
tel: + 33 (0) 3 88 27 91 73 - fax: + 33 (0) 3 88 27 91 41

5.3 Conditions of storage and use

The study drug TG4010 must be stored at or below -70°C in a freezer under the supervision of the study pharmacist / investigator. The ampoules will be dispensed only with the written authorization of the investigator to staff that have been specifically designated to this study.

5.4 Preparation for administration

TG4010 is a Genetically Modified Organism (GMO). TG4010 must be handled and administered to patients under the conditions which should be recommended by the corresponding national/local regulatory authorities based on the European Directives 90/219 and 90/220 related to manipulation and dissemination of Genetically Modified Organisms.

A Technical Sheet and a detailed Preparation Procedure of the study drug will be provided to the study pharmacist / investigator for each lot to be used.

All transfers of the preparation must be done using a closed container.
During the study drug manipulations specific protective clothing must be worn (**labcoat, gloves and goggles)**. Prior to the administration, the study drug must be prepared under aseptic conditions.
Once prepared, the study drug must be maintained between +2°C and +8°C. The administration should not take place later than 4 hours after the dispensation.

In case of incident with the use of the TG4010, please act as recommended below:
- Accidental shedding:
Contaminated area must be cleaned with a disinfectant active on the study drug or bleach (≥ 1.6°Cl i.e. 5 g/l of active chlorine).
- Needlestick injury:
Wash immediately under tap water. Then treat the area as follows:

- Wash with soap for 5 minutes. Rinse. Then treat the area with bleach (≈ 1.4°Cl i.e. 4.5 g/l of active chlorine) for 5 minutes. Rinse again.

or:

- Wash with a solution of 4% iodine for 5 minutes. Rinse. Then treat the area with a solution of 10% iodine for 5 minutes. Rinse again.

Cover with an occlusive, dry dressing, which should be appropriately discarded when removed. The injured person should receive counselling from the investigator and should then be closely followed for a period of at least 2 weeks.
- Eye contamination:
Irrigate immediately the eye during 15 minutes with lukewarm water being careful not to contaminate the other eye. Instil one drop of a solution of trifluridine 1%. The injured person should receive counselling from an ophthalmologist as soon as possible.
- Ingestion:
Do not induce vomiting and call the investigator or a doctor immediately. The person should be closely followed for a period of at least 2 weeks.
All incident must be documented by a written report immediately sent to Transgene Medical Affairs (Fax: + 33 (0)3.88.27.91.41)

Recommendations for the decontamination and the destruction of the material used for the study drug preparation and administration are available in the Investigator's Brochure and in the Preparation Procedure.

6 TREATMENT PLAN

6.1 Treatment administered

6.1.1 Treatment regimens

Patients will be randomized to one of the following two treatment regimens:

- **Arm 1 (TG4010 - combination chemotherapy):**
  - Patients randomized into this arm will receive SC injections of TG4010 at the dose of 10^8 pfu, once every week for 6 weeks then once every 3 weeks, on days 1, 8, 15, 22, 29, 36, 43, 64, 85, 106, 127, etc. until documentation of progressive disease.
  - Patients randomized into this arm will also receive chemotherapy with cisplatin and gemcitabine on a 3 weeks schedule for up to 6 cycles or until documentation of progressive disease.
  - A biological threshold for administration of TG4010 at days 15 and 36 where TG4010 is given without chemotherapy is defined as: neutrophils: $0.5x10^9$/L.
    The administrations are cancelled but not delayed. The next administration is to be done at the first day of a new course of chemotherapy.

- **Arm 2 (chemotherapy alone):**
  - Patient randomized into this arm will receive chemotherapy with cisplatin and gemcitabine on a 3 weeks schedule up to 6 cycles or until documentation of progressive disease.
  - The patient will be followed until documentation of disease progression.

- **For both arms:**
  - The chemotherapy dose and schedule are the following:
    - Cisplatin: 75mg/m² at day 1,
    - Gemcitabine 1250mg/m² at days 1 and 8,
    - New course of chemotherapy at day 22.
  - According to standard practice, chemotherapy will be delayed if the recovery from adverse events (i.e. hematological toxicity) associated with the previous cycle of chemotherapy does not allow the administration of a new cycle. In this case, all actions planned on this day and all following assessments will be delayed by one week, including the TG4010 injection for patients of arm 1.
  - The hematological threshold for administration of chemotherapy in this study is defined as:
    - Neutrophils: $1.5x10^9$/L for day 1 of each course of chemotherapy
      $1.2x10^9$/L for day 8 of each course of chemotherapy
    - Platelets: $100x10^9$/L

Cisplatin may be replaced during study by an adequate dose of carboplatin for a given patient only in two situations:

- Cisplatin induced ototoxicity documented by an audiogram;

- Cisplatin induced nephrotoxicity documented by a reduction of creatinin clearance below 60 ml/min.

Doses of cisplatin and/or gemcitabine may be reduced during study for a given patient according to standard practice of each investigational center, in the case of bad tolerance.
Both these therapeutic adaptations need to be documented in the CRF.

6.1.2 Administration of the study drug

TG4010 will be administered at the dose of 10^8 pfu by SC injection in a single injection. The volume to be injected may vary from one lot to another but will not exceed 1 ml. The precise volume is indicated on the preparation procedure supplied along with the study drug.

Four injection sites will be used: left and right arm, left and right thigh, according to a rotation schedule. It is preferable that the first vaccination be given in either the right or left thigh. The second vaccination would be given in the opposite side arm, third in the same side thigh and fourth in the opposite side arm. As an example, if the left thigh is the site of the first vaccination, the second vaccination would be in the right arm, third the right thigh and fourth the left arm. This same pattern would be repeated for subsequent vaccinations. If the pattern cannot be followed exactly, it is more important to alternate between the arms and thighs than side to side.

Patients will be monitored for half an hour after each study drug injection. For this reason it is more convenient to inject TG4010 during the preliminary hydration associated to the chemotherapy. Regarding the Genetic Modified Organism (G.M.O.) use (manipulation and patient administration) National Regulatory requirements will be applied.

6.1.3 Duration of treatment and observation period

The duration of treatment and observation lasts from the first injection until 4 weeks after documentation of progressive disease.
Patient will be followed off study for documentation of long-term clinical evolution (further treatments after withdrawal, response to treatments, and survival) on a 3 months basis.

6.2 Method of assigning patients to treatment groups

6.2.1 Recruitment and Randomization

The investigator will be asked to complete a “screening log” in which each potential patient (i.e. screened patient) will be listed. After having signed the informed consent form, a screened patient is considered as a “consented patient”.
Upon satisfaction of all of the inclusion and exclusion criteria of a consented patient, the site completes a randomization form and faxes it to the CRO.
Once the CRO is informed by fax that a patient fulfils all inclusion criteria, it will send back to the centre the same form completed with both the assigned treatment arm and the patient number in the study. After this step the patient is considered as an “included patient”.
This process is fully documented on the “screening log” should a patient or not being included in the study.
Under no circumstances will a patient entered in the study be permitted to re-enroll for a second time in the study.

The randomization list will be generated by the statistician prior to the start of the study. It will be a standard block design comprised of a block size of four for each centre.

6.2.2 Stratification / Minimizations

Patients will be centrally allocated to one of the two treatment arms (cisplatin-gemcitabine or TG4010 with cisplatin-gemcitabine) using a dynamic minimization procedure using center and the following factors that are known to be of prognostic importance in advanced non small cell lung cancer:

- Stage (IIIb versus IV),
- Performance status (0 versus 1).

The dynamic minimization will use a stochastic treatment allocation algorithm based on the variance method {Freedman LS, White SJ, 1976}.

6.2.3 Dose modifications

No dose modifications of TG4010 are permitted.

6.3 Treatment compliance, dispensing and accountability

The study drug will only be dispensed, according to investigator's prescription, to patients who meet all selection criteria.

The investigator / study pharmacist will maintain a study drug accountability log detailing the dates and quantities dispensed for each patient along with ampoules and lots numbers. Study drug accountability records will be verified by the monitor during site visits. All used and unused study drug will be accounted for. All unused study drug will be returned to the sponsor or destroyed locally at the end of the study. The investigator / study pharmacist will ensure that this alternative disposition is performed according to Transgene's instructions and will not expose human to risks from the study drug. Moreover a "certificate of destruction" will be established.

The prescription and tracking form for study drug will also be used to track all study drug during the preparation process from prescription to destruction (through dispensation, reconstitution, administration and decontamination).

6.4 Premature withdrawal of patients

6.4.1 Circumstances

The patient's participation in the protocol will terminate under any of the following circumstances:

- Patient request at any time for any reason,
- Physician determination that patient's further participation in the protocol is not in the patient's best interest.
- At the determination of the sponsor (for example in case of new fact or toxicity issue regarding the study drug).
- Patients who have severe toxicity (Grade 3, 4) associated with the study drug.
- Progressive disease as defined in section 8.1.

For any discontinuation, the investigator will obtain all the required details and document the date of and the reason for the discontinuation in the CRF. In any case of treatment cessation not related to disease progression the patient will be followed per protocol until documentation of progressive disease.
If the reason for stopping the treatment is an AE, the specific event or the main laboratory abnormality will be recorded in the CRF. The investigator will make thorough efforts to document the outcome.
As far as possible, no patient should leave the study without having undergone the end of study visit.

6.4.2 Replacement policy

Whatever the reason for withdrawal, patients considered as non-evaluable for the purpose of the study will be replaced. This concerns only non progressing patients lost to follow-up within the 6 months following randomization.

7 STUDY VISITS AND PROCEDURES

A flow-chart, shown in appendix 1, summarizes the evaluations to be performed and their time points. If not otherwise specified, when an assessment is planned on the same day as an injection, it will be done prior to the injection.

7.1 Evaluations description

Clinical evaluation:
Interim history and physical examination of the major organ system, including vital signs and weight.
Performance status on ECOG scale.
Patients will be monitored before and for half an hour after each study drug injection.

Safety assessment:
The reporting, assessment and follow-up of all pre-drug events (PDEs) and adverse events (AEs) will be documented in the CRF.

Quality of life assessment:
Evaluation of quality of life with a FACT-L form to be completed by the patient {Cella D.F., 1995}.

Hematology:
Complete blood count including RBC, hemoglobin, WBC and differential, platelets.

Laboratory evaluation:
- Liver function tests: bilirubin, ALT, AST, γGT, PAL and LDH;
- Renal function tests: creatinine;
- Coagulation parameters: D-dimers, PTT;
- Inflammatory parameters: CRP;
- Glycemia;

- Electrolytes: Na^+, K^+, Ca^{++};
- CD4+ count;
- Proteinemia, albuminemia.

Tumor markers:
CA15-3, CEA, CA19-9, CA125 and CYFRA21-1 at baseline. Only those markers elevated at baseline will be followed during study.

Tumor evaluation:
Documentation of known sites of metastatic disease, other diagnostic evaluations as clinically indicated; all measurable and non measurable lesions must be assessed using the same techniques as baseline. Results must be available prior to the planned visit, to decide whether the patient should continue the treatment or not.

Cardiac evaluation: Electrocardiogram at baseline.

Pulmonary evaluation: Chest X-Ray at baseline.

Immuno-histology evaluation:
Histological documentation of MUC1 antigen expression on the primary tumor or on metastasis, as defined by a positive staining by immuno-histo-chemistry in at least 25% of the tumor cells in the conditions described in the technical documentation of the monoclonal antibody;

Auto-immunity parameters: Anti-nuclear and Anti-TPO antibodies.

Immunology evaluation:
WBC sampling for T and NK cell specificity and phenotype analysis (tetramers and flow cytometry).

Proteomic analysis: Plasma sampling for proteomics by mass spectrometry. Remaining samples of patient serum or plasma will be stored for other translational research techniques involving plasma or serum components which may become available and may be deemed important for understanding the mechanism of action of TG4010 or associated toxicities.

7.2 Baseline

Prior to initiation of treatment (All evaluations must be performed within 21 days prior to the initiation of treatment):

- Documentation of histological diagnosis, including positive staining for the MUC1
- Clinical evaluation with complete medical history and physical examination, including history of prior treatment of lung cancer, symptoms attributable to tumor and performance status (ECOG scale).
- FACT-L
- Cerebral CT-scan or MRI
- In case of bone pain, an adequate radiological and/or isotopical exploration is to be performed to research metastasis
- Tumor evaluation (report of all lesions)
- Chest X-Ray

- Electrocardiogram
- Tumor markers: CA15.3, CEA, CA19.9, CA125 and CYFRA21-1
- Laboratory evaluation and hematology
- Anti-nuclear and Anti-TPO antibodies
- Immunology evaluation
- Proteomic analysis
- HLA typing (MHC Class I only)
- Detection of antibodies against HIV; detection of antigen HBs and HCV serology
- Pregnancy test (for child bearing potential women)

7.3 While enrolled on the protocol

7.3.1 First 6-week period

- **Day 1:**
    - Clinical evaluation including vital signs to be repeated thirty minutes after TG4010 injection for patients treated in arm 1
    - Safety assessment
    - Laboratory evaluation and hematology
    - Initially elevated markers
    - Chemotherapy: cisplatin: 75mg/m² + gemcitabine 1250mg/m²
    - TG4010 injection for patients treated in arm 1

- **Day 8:**
    - Clinical evaluation including vital signs to be repeated thirty minutes after TG4010 injection for patients treated in arm 1
    - Safety assessment
    - Hematology
    - Chemotherapy: gemcitabine 1250mg/m²
    - TG4010 injection for patients treated in arm 1

- **Day 15: only for patients treated in arm 1**
    - Clinical evaluation including vital signs to be repeated thirty minutes after TG4010 injection
    - Safety assessment
    - Hematology
    - TG4010 injection

- **Day 22:**
    - Clinical evaluation including vital signs to be repeated thirty minutes after TG4010 injection for patients treated in arm 1
    - Safety assessment
    - Laboratory evaluation
    - Hematology
    - Initially elevated markers
    - Chemotherapy: cisplatin: 75mg/m² + gemcitabine 1250mg/m²
    - TG4010 injection for patients treated in arm 1

- **Day 29:**
  - Clinical evaluation including vital signs to be repeated thirty minutes after TG4010 injection for patients treated in arm 1
  - Safety assessment
  - Hematology
  - Chemotherapy: gemcitabine 1250mg/m²
  - TG4010 injection for patients treated in arm 1

- **Day 36: only for patients treated in arm 1**
  - Clinical evaluation including vital signs to be repeated thirty minutes after TG4010 injection
  - Safety assessment
  - Hematology
  - TG 4010 injection

7.3.2 Subsequent 6-week periods

- **Days 43, 85, 127, 169, etc (first day of a new 6-week protocol cycle):**
  - Clinical evaluation including vital signs to be repeated thirty minutes after TG4010 injection for patients treated in arm 1
  - Safety assessment
  - Tumor evaluation, as for baseline (ideally within a period of not more than 5 days prior to study visit)
  - FACT-L
  - Laboratory evaluation
  - Hematology
  - Initially elevated markers
  - Anti-nuclear and Anti-TPO antibodies
  - Immunology evaluation (only at days 43 and 85)
  - Proteomic analysis (only at days 43 and 85)
  - Chemotherapy up to 6 cycles or until documentation of progression disease cisplatin: 75mg/m² + gemcitabine 1250mg/m²
  - TG4010 injection for patients treated in arm 1 until documentation of progressive disease

- **Days 50, 71, 92, 113 up to 6 cycles of chemotherapy or until documentation of progressive disease**
  - Clinical evaluation
  - Safety assessment
  - Hematology
  - Chemotherapy: gemcitabine 1250mg/m²

- **Days 64, 106, 148, 190, etc.**
  - Clinical evaluation including vital signs to be repeated thirty minutes after TG4010 injection for patients treated in arm 1
  - Safety assessment
  - Laboratory evaluation
  - Hematology
  - Initially elevated markers

- Chemotherapy up to 6 cycles or until documentation of progression disease cisplatin: 75mg/m² + gemcitabine 1250mg/m²
- TG4010 injection for patients treated in arm 1 until documentation of progression disease

7.4 End of study visit

The following evaluations will be conducted upon withdrawal from the protocol and at least 4 weeks after the last TG4010 or chemotherapy injection:

- Clinical evaluation including vital signs
- Safety assessment
- FACT-L
- Laboratory evaluation and hematology
- Initially elevated markers
- Anti-nuclear and Anti-TPO antibodies

7.5 Post-study follow-up

Patient will be followed off study for documentation of long-term clinical evolution (tumor response, further treatments after withdrawal and survival) on a 3 months basis. A specific page for follow-up will be supplied in the CRF and be sent to Trangene once completed.

7.6 Handling of biological samples

See Laboratory Manual.

8 ASSESSMENT OF EFFICACY

8.1 Primary variable

The primary variable will be the progression free survival at 6 months. The progression at any moment is defined according to WHO criteria.

WHO criteria: sum of the maximum cross-sectional areas (determined by the product of the maximum diameter of a tumor and the largest diameter perpendicular to this maximum diameter). All measurable and non-measurable lesions must be assessed using the same techniques throughout the study.

Complete Response (CR): Complete disappearance of all measurable and non-measurable disease, no new lesions, no residual disease related symptoms, no evidence of non-evaluable disease. This must be maintained for a minimum of four weeks.

Partial Response (PR): ≥ 50% decrease in the sum of products of perpendicular diameters of all measurable lesions, no progression of evaluable disease, and no new lesions, maintained for a minimum of four weeks. Non-measurable lesions must remain stable or regress during this time.

Progressive Disease (PD): A ≥25% increase in the sum of products of all measurable lesions over the smallest sum observed, using the same techniques as baseline, or clear worsening of any non-measurable disease or reappearance of any lesion which had previously disappeared, or appearance of any new lesion or site of disease, or overall clinical deterioration or death attributable to cancer.

No Change: neither PR nor PD criteria met.

8.2 Secondary variable(s)

- Response rate: according to WHO criteria (see above).
- Time to Progression: Measured from the date of randomization to the first date the patient satisfies to the definition of a progressive disease.
- Overall survival: Measured from the date of randomization to the date of death.

8.3 Appropriateness of measurements

PFS at six months being the primary endpoint of the study, a central review of the tumor evaluations, blinded for the treatment arm, is planned for all the patients not progressive at 6 months or responding to therapy. For this purpose an anonym duplicate of all the radiological documents evaluating the tumor will be collected. The radiological folder comprises the radiological examinations from baseline to progression as well as the first subsequent tumor evaluation performed after study withdrawal whatever the treatment the patient may have received. The date of progression stated by the central review is taken into account for the determination of the PFS. In case of discrepancy a second central lecture is realized.

8.4 Review of the pathological samples

Depending on the conclusions of the study, a review of the pathological samples used for MUC1 staining may be organized.

9 ASSESSMENT OF SAFETY

The condition of the patient will be monitored throughout the study.
Overall incidence of adverse events and serious adverse events will be evaluated for each arm of treatment and for the study as a whole.

9.1 Definitions

Adverse Event (AE)
Any untoward medical occurrence in a patient or clinical investigation subject administered a pharmaceutical product and which does not necessarily have a causal relationship with this treatment. An AE can therefore be any unfavorable and unintended sign (including an abnormal laboratory finding), symptom, or disease temporally associated with the use of a medicinal (investigational) product, whether or not related to the medicinal (investigational) product.
An AE is therefore regarded as such only if it starts **after the first administration of the study drug**.

<u>Pre-drug event (PDE)</u>
Any event occurring before the first study drug administration, i.e. between the patient signature of the informed consent form and the first administration of the study drug.

<u>Laboratory abnormality</u>
A laboratory abnormality is reported as an AE if it is out of range, considered by the investigator as clinically significant (i.e. with clinical manifestations or requiring treatment or clinical management) and confirmed by a repeat measurement (if relevant). Worsening from pre-drug administration state are considered on the same basis.

<u>Other significant adverse events</u>
Any events and any laboratory abnormalities that led to an intervention, including withdrawal / dose reduction of study drug or significant additional concomitant therapy other than those reported as SAE, and that are considered by Transgene or the investigator to be of special interest because of clinical importance.

<u>Serious Adverse Event (SAE)</u>
Any untoward medical occurrence that at any dose:

- results in death,
  The death of a patient is not per se an AE but an outcome. “Death” should be considered as a SAE only in case of “unexplained death" when no cause is identified. The event that resulted in a fatal outcome should be determined.
- is life threatening,
  This term refers to an event in which the patient was at immediate risk of death at the time of the event; it does not refer to an event that hypothetically might have caused death if it was more severe.
- requires inpatient hospitalization or prolongation of existing hospitalization,
  The hospitalization is an action taken to treat the event. It should not be reported as a SAE, but the AE leading to hospitalization.
  Hospitalization for diagnosis or planned treatment procedures without AE should not be reported as a SAE.
- results in persistent or significant disability/incapacity,
  The disability is a substantial disruption of a person’s ability to conduct normal life functions.
- is a congenital anomaly/birth defect,
- is an important medical event that may not be immediately life threatening or result in death or hospitalization, but may jeopardize the patient and may require medical or surgical intervention to prevent one of the outcomes listed above (example: intensive treatment in an emergency room or at home for bronchospasme, convulsions that do not result in hospitalization…). Medical and scientific judgment should be exercised in deciding whether some events should be considered as serious because their quick reporting to the sponsor may be of interest for the overall conduct of the study.

<u>Serious Pre-drug events (SPDEs):</u>
Any serious events occurring before the first study drug administration, i.e. between the patient signature of the informed consent form and the first study drug administration.

<u>Overdose</u>
An overdose is an administration of a study drug at a higher dose than the highest dose already tested in clinical studies or higher than known therapeutic doses.

<u>Verbatim</u>
The terms or the English equivalent of the terms as reported by the investigator on the CRF pages and on the SAE form to describe an event.

9.2 Intensity, relationship and outcome evaluation

<u>Intensity</u>
The intensity of the clinical adverse event is graded according to the Common Terminology Criteria for Adverse Events (CTCAE) version 3.0 which is provided to the investigators. Should an event be missing in the CTCAE, the following 3 point scale is used:

- Mild: Discomfort noticed, but no disruption of normal daily activity.
- Moderate: Discomfort sufficient to affect normal daily activity.
- Severe: Inability to work or perform normal daily activity.

The correspondence between the two scales is as follows:

**CTCAE**	**3 point scale**
1	Mild
2	Moderate
3 / 4 / 5	Severe

<u>Relationship to the study drug</u>
The relationship is evaluated as follows:

- Unrelated: There is evidence of relationship to a cause other than the study drug. Does not meet criteria listed under unlikely, possible or probable.
- Unlikely: Does not follow a reasonable temporal sequence from administration. Is most likely produced by the patient's clinical state or by environmental factors or other therapies administered.
- Possible: Follows a reasonable temporal sequence from administration. Is not likely produced by the patient's clinical state or by environmental factors or other therapies administered.
- Probable: Follows a reasonable temporal sequence from administration. Clear-cut temporal association with improvement on cessation of study drug. Reappears upon re-challenge.

<u>Outcome</u>
The outcome is rated as follows:

- recovered,
- not recovered,
- recovered with *sequelae* (to be specified on comment page),
- fatal,
- unknown,
- worsening.

Note on "fatal": this outcome is to be used only for the event leading to death. The outcome of all other events at the time of the death must be reported. The outcome of ongoing ones is reported as "not recovered".

Note on “worsening”: this outcome is used when a PDE worsens and becomes an AE or when an AE worsens. The new status of the event is documented on a second line of the PDE or AE page in the CRF.

9.3 Pre-drug event management

Reporting in CRFs
Any PDE directly observed or mentioned by the patient before the first study drug administration will be reported by the investigator or designee on the page "Events occurring before the first study drug administration" of the CRF (= PDE page). The following items must be documented:

- nature of the event with self explanatory and concise medical terminology (indicate a diagnosis or syndrome instead of symptoms),
- date of onset and date of end (i.e. actual dates when the event starts and is resolved rather than dates when the investigator is informed),
- outcome,
- intensity,
- action taken regarding the study,
- action taken regarding the event,
- evaluation of seriousness.

PDEs requiring therapy must be treated with recognized standards of medical care to protect the health and well being of the patient. Any treatment given will be reported on the page "Concomitant medication" of the CRF.
Appropriate resuscitation equipment and medicines must be available to ensure the best possible treatment of an emergency situation.

Follow-up
If those events are not resolved before the first study drug administration they will be followed until resolution or the last visit planned by the protocol:

- PDE with no change will not be considered as AE. The outcome is documented when known. If a PDE is still ongoing at the last study visit, the status at this time will be documented
- PDE that worsens after the first study drug administration will be considered as AE and managed as follows in the CRF:
  - On the PDE page, the line of the PDE is completed with the outcome ticked as “worsening”.
  - On the AE page, a new event is reported and managed as described in section 9.4. The wording of the event should include "worsening of" or something similar.

It is possible that, due to its nature or intensity, a PDE could delay the first study drug administration or lead to patient withdrawal. This will be documented on the PDE page of the CRF in the column “Action taken regarding the study”.

Documentation
PDEs will be reported in the patient source document with at least the nature, the start date and the treatment (if applicable).

PDE pages of the CRF will be signed by the investigator once fully completed.

9.4 Adverse event management

Reporting in CRFs
At each visit, any AEs directly observed or mentioned by the patient will be reported by the investigator or designee on the page “Adverse Events” of the CRF (= AE page). The following items must be documented:

- nature of the event with self explanatory and concise medical terminology (indicate a diagnosis or syndrome instead of symptoms),
- date of onset and date of end (i.e. actual dates when the event starts and is resolved rather than dates when the investigator is informed),
- outcome,
- intensity,
- relation to study drug,
- action taken regarding the study drug,
- action taken regarding the event,
- evaluation of seriousness.

AEs requiring therapy must be treated with recognized standards of medical care to protect the health and well being of the patient. Any treatment given will be reported on the page "Concomitant medication" of the CRF.
Appropriate resuscitation equipment and medicines must be available to ensure the best possible treatment of an emergency situation.

Grade 3/4 Adverse Events
All grade 3/4 AEs related to the study drug and any other relevant AE related to the study drug which do not qualify as a SAE, will be further documented on "additional information on adverse events" CRF page and faxed to Transgene.

Follow-up
AEs must be followed until resolution or the last visit planned by the protocol.
The AEs listed below must be followed until they are resolved or stable, which may occur after the last visit planned by the protocol:

- AEs with a possible/probable relationship with the study drug,
- AEs leading to withdrawal from the study,
- Any other significant adverse event.

Documentation
AEs will be reported in the source document with at least the nature, the start date and the treatment (if applicable) of the event.

AE pages of the CRF will be signed by the investigator once fully completed.

9.5 Serious adverse event management

Reporting to Transgene

Any SAE occurring during the course of a study, i.e. between the first study drug administration and the end of study visit, irrespective of the treatment received by the patient MUST be reported to Transgene. The investigator must complete and fax a "Serious Adverse Event Form" to Transgene within ONE WORKING DAY of occurrence or knowledge of the event.

	**France**
	**Safety Officer**
**Name**	I. DIDILLON
**Phone number**	+ 33.(0)3.88.27.91.73
**Fax number**	+ 33.(0)3.88.27.91.41
**Emergency 24-hour telephone number**	+ 33.(0)3.88.27.91.73

An investigator designee may complete the SAE form, however, the investigator must sign it. The form can sent to Transgene with the designee's signature if the investigator's signature cannot be obtained within one working day. The investigator's signature must be obtained as soon as possible, as well as his/her evaluation of the relationship to the study drug. The signed form must be faxed to Transgene immediately.
As far as possible the SAE form should be completed in English.

Special cases

The following events will be documented, reported and followed-up in the same way as SAEs:

- Serious PDEs,
- Overdose (even if no toxic effects are observed),
- Pregnancy (to be followed up in the same way as a SAE until the final outcome of the pregnancy and knowledge of the new-born medical status).

Follow up

If follow-up information is not available at the time of the event, this information must be forwarded as soon as possible to Transgene using a new SAE form with the box "follow-up" ticked. New information must be sent to Transgene within one day of knowledge. Transgene may request information as needed.
All SAEs will be followed until the final outcome is known.

Post study SAE

Any SAE occurring after the end of study visit **and** that is considered by the investigator to be possibly or probably related to the study drug must be reported to Transgene, documented and followed-up as described above.
If the end of study visit is performed less than 28 days after the last study drug administration, ALL SAEs occurring between the end of study visit until 28 days after last study drug administration will be considered as SAE and notified to Transgene, regardless of their relationship to study drug.

Notification to Regulatory Authorities / Gene Therapy Bodies / Ethics Committees
Transgene is responsible for notifying SAEs to Health Authorities and to the IEC (Independent Ethics Committee) in accordance with local regulation. Gene Therapy Bodies are informed as locally required.

Information to investigator
When a serious, unexpected, probably or possibly related event has been notified to Health Authorities, Transgene will inform all other investigators working in this study as well as those working with the same study drug in other studies.

Documentation
All SAEs will be reported on the AE pages of the CRF using the same information as documented on the SAE form and in source documents as described in §9.4. Copies of SAE form will be filled in the Investigator Site File along with copies of any correspondence with the IEC. The Investigator Site File will also include copies of notification letters and/or faxes with forms sent to Health Authorities and Gene Therapy Bodies if appropriate.

9.6 Laboratory values, vital signs, physical findings and other safety data

Clinically relevant abnormal laboratory results will be repeated immediately (if relevant) and followed until return to normal unless an adequate explanation is determined.

Clinically relevant abnormal findings following vital signs measurements or physical examinations will be reported as AEs.

10 STATISTICAL METHODS PLANNED AND SAMPLES SIZE

10.1 Determination of sample size

The purpose of this trial is to screen for activity of the combination of TG4010 with cisplatin-gemcitabine. Since TG4010 may not induce tumor shrinkage but may prevent or delay tumor growth, activity is evaluated in terms of time to progression, with the decision rule of the trial being based on the proportion of patients who are still free of progression 6 months after starting therapy. The study is designed as a one-stage phase IIb trial with the following assumptions {Fleming T.R., 1982}:

- the inactivity cut-off is chosen equal to 30%, the activity cut-off equal to 50%. Hence the hypotheses of interest are H_0: $r \leq 30\%$ against H_A: $r \geq 50\%$, where r is the proportion of patients who are free of progression at 6 months
- the type I error rate (α, probability of accepting an insufficiently active treatment, a false positive outcome) is set to 5%
- the type II error rate (β, probability of rejecting an active treatment, a false negative outcome) is set to 5%

Under these assumptions, the design consists of the following decision rule: treat 67 evaluable patients.

- if at most 26 patients are alive and free of progression at 6 months, declare the treatment inactive and stop the randomised trial.

- if at least 27 patients are alive and free of progression at 6 months, declare the treatment active.

Assuming that less than 5% of the patients will discontinue the study before disease progression, a total of 140 patients must be randomised (70 per treatment arm, to produce 67 evaluable patients by treatment arm).

10.2 Continuation of trial in phase III

If the treatment is declared active according to the phase IIb decision rule, the trial may be continued as a phase III trial. An appropriate sample size for the phase III trial will then be calculated, based on external information (other trials and published references), not on the results of the phase IIb portion of the trial. The phase II portion of the trial is neither aimed at, nor powered for, the detection of a difference in response rate, time to progression or survival between the two randomised arms. The phase IIb portion of the trial will not be considered as constituting an interim analysis of the phase III trial, and therefore the significance level of the tests at the end of the phase III trial will not require adjustment (unless proper interim analyses of the phase III trial are later conducted).

10.3 Study endpoints

Primary endpoint

Progression free survival (PFS) at 6 months. Progression free survival will be calculated from the date of randomization to the date of disease progression or to the date of death from any cause.

Secondary endpoints

Response rate will be defined according to the WHO criteria.

Duration of response will be defined for all evaluable patients who have achieved an objective response from the date at which the patient's objective status is first noted to be a complete or partial response to the date progression is documented (if one has occurred) or to the date of last follow-up (for those patients who have not progressed).

Overall survival will be defiened from the date of randomization to the date of death from any cause

Time to progression will be defined from the date of randomization to the date of disease progression or death from malignant disease.

Safety assessed by the incidence of adverse events, serious adverse events, and number of study discontinuations

Cellular immune response will be assessed by tetramer and immunophenotype analysis.

Plasma proteomic profile will be assessed by mass spectrometry.

10.4 Statistical and analytical plan

Estimation

All proportions will be estimated with their exact (binomial) 95% confidence intervals.

The distribution of time to event variables (progression free survival, time to progression, overall survival and duration of response) will be estimated using the Kaplan-Meier method.

Statistical tests

Efficacy and toxicity endpoints will be compared using the following tests, stratified for tumor stage and perfomance status:
- the logrank test for time to event variables
- the Cochran-Mantel-Haenszel chi-square test for proportions (response rates and adverse events).

Safety analyses

The safety data will be classified according to their intensity and relationship to the study drug. The more common AEs, laboratory test changes, etc. will be identified, classified and analyzed, as appropriate, for factors that may affect the frequency of AEs, such as time dependence, relation to demographic characteristics or relation to dose. The number of patients with each AE will be displayed by decreasing frequency of occurrence and body system classes. All AEs occurring after the initiation of the study treatments, including events likely to be related to the underlying disease or likely to represent concomitant illness should be reported, including events present at baseline which worsened during the study. Written narratives will be provided for all serious, unexpected or other important AEs that are judged to be of special interest because of their clinical importance.

10.5 Disposition and replacement of patients

The number of patients screened and not included will be presented with the main reason for their non-inclusion. All patients who failed to meet inclusion criteria after informed consent signature will be considered as screening failure and reported in the study report.
All patients randomized and who completed each step of the study will be considered in the study report. All post-randomization discontinuations will be summarized by main reason for discontinuation.
The decision rule being based on an absolute number of patients evaluable until 6 months, patients who discontinue the study before disease progression will be replaced so as to reach the required number of evaluable patients.

10.6 Protocol deviations

Any protocol deviation will be discussed with the investigator on a case by case basis and documented in the CRF on “Additional comments” pages.

10.7 Data sets analyzed

All patients entered into the study who received at least one dose of study drug will be included in the safety analysis.

Patients who are evaluable at 6 months (all randomized patients less those who discontinue the study prior to disease progression or death) will be included in the decision rule for treatment activity.

All other analyses will be based on intention to treat, i.e. on all randomized patients.

11 CHANGES IN THE CONDUCT OF THE STUDY

11.1 Protocol amendments

Changes to this protocol will be effected through amendments issued by Transgene after mutual agreement of the investigator(s) and Transgene. Both the investigator(s) and Transgene will sign the amendments. Amendments are submitted to Health Authorities and the Independent Ethics Committee (IEC) and any other committees by Transgene or the investigator according to local regulations.

Ethical approval will be required for any change to the protocol which could significantly affect the safety of patients, the scope of the investigation or the scientific quality of the study. Other changes will be provided to IEC(s) and any other committees for information only.

IEC(s) and any other committee’s approval must be obtained before implementation of change(s) except where necessary to eliminate immediate hazards to the patients or when the change(s) involves only logistical or administrative aspects of the study.

11.2 Premature study termination

Both the investigator and the sponsor reserve the right to terminate the study at any time. Should this become necessary, the procedures will be agreed upon after consultation between the two parties. If the study needs to be terminated, Transgene and the investigator will assure that adequate consideration is given to the protection of the patients.

Transgene will notify the Health Authorities and the IEC and any other committees of the premature study termination according to local regulations.

12 ETHICAL CONSIDERATIONS

12.1 Independent Ethics Committee

Before starting the study, the protocol, the written patient information sheet and informed consent form, and any other document specifically requested must be reviewed and approved by an IEC complying with the requirements of relevant local law.

A written approval and the list of members who participated in the meeting must be obtained by Transgene / the investigator before enrollment of patients.

In addition, IEC written approval must be obtained by Transgene / the investigator for any protocol amendment.

12.2 Informed consent

The investigator will obtain a voluntary written consent from each patient after an appropriate explanation of the aims, methods, anticipated benefits, risks and any other aspect of the study relevant to the patient’s decision to participate. Consent forms and all verbal study related information must be in a language fully comprehensible to the prospective patient.

Patients will be informed that they are free not to participate in the study and that they may withdraw consent to participate at any time. They will be told which alternative treatments are available if they refuse to take part and that such refusal will not prejudice future treatment.

Patients will be informed that their records, including medical history, may be examined by competent authorities and authorized persons but that personal information will be treated as strictly confidential and will not be publicly available.

The “patient information sheet” will explain that the data collected for this study will be stored in a computer database, with confidentiality maintained in accordance with national data legislation. All data computer processed will be identified by patient initials and number only.
A written “patient information sheet” will be given to each patient to complete the verbal information. This written form should be reviewed orally with the patient. Patient must be given ample opportunity to inquire about details of the study.

Informed consent shall be documented by the use of a written consent form approved by the IEC and signed and dated by the investigator and the patient before any exposure to a study-related procedure, including screening tests for eligibility.

For patients unable to give a legally valid consent, the written informed consent must be obtained from the patient’s legally authorized representative. The legally authorized representative must undergo the entire informed consent process with the patient, sign and personally date the informed consent form. The patient should be informed about the study to the level of his/her understanding and should, if capable, sign and personally date the written informed consent form. Further, the reason for being unable to obtain standard consent is to be documented in the patient’s source document.

Should a patient start with the study drug treatment more than 1 months after having signed the informed consent form, whatever the reason, it would be ethical to ask him/her to reconfirm his/her willingness to participate to the study by signing a new consent form.
A copy of each signed informed consent form must be given to the patient and to his/her legally authorized representative. The originals are filed at the study site in the investigator site file.

12.3 Confidentiality of patient data

The investigator must assure that patients' anonymity is maintained and that their identities are protected from unauthorized parties. On CRFs or other documents collected by Transgene or its representative, patients should not be identified by their names, but by an identification code system consisting of their initials and a number.

The investigator should keep a patient identification log showing codes, names and addresses of all patients consented. A copy of this log without names and addresses will be filed at Transgene after study completion.

13 REGULATORY CONSIDERATIONS

13.1 Regulatory considerations

This study will be conducted in accordance with:

- The updated Declaration of Helsinki adopted by the World Medical Association,
- The ICH (International Conference on Harmonization) Good Clinical Practice (GCP) guidelines, and
- The local regulatory requirements.

13.2 Regulatory approval / authorization

The regulatory permission for conducting the study will be obtained in accordance with local regulatory requirements. All approvals must be obtained before a patient is exposed to a study-related procedure, including baseline screening tests for eligibility. Additional approvals will be obtained from the national gene therapy and viral safety committees, as required.

13.3 Investigators obligations

Before the study starts, the investigator shall supply Transgene with his/her curriculum vitae and completes a list giving the names, functions and authorized activities of all persons who will exercise any kind of responsibility in carrying out of the study.

The investigator ensures the quality of the study through strict observance of the protocol, Good Clinical Practice and local regulations. Investigator must ensure that the study has been approved by all required IEC and any other committees prior to enrolling patients and on an ongoing basis as locally required. Investigator is required to obtain written informed consent from each patient prior to study entry.

The investigator also provides to the site staff appropriate training. The staff training will be documented in the investigator site file and a copy of this document will be provided to Transgene.

13.4 Insurance

Transgene certifies having taken out a civil liability insurance policy covering liability with regard to the participants in this study.

Insurer: Chubb
Policy no: 600 728

14 QUALITY CONTROL AND QUALITY ASSURANCE

14.1 Source data and documents

Source data are all information available in original source document or certified copies of source document of any clinical findings, observations, or other activities that are necessary for the reconstruction and evaluation of the study.

The investigator will record the following information in the source documents for all patients enrolled, including but not limited to: patient name, date of birth, sex, medical history, information that the patient is included in the study, visit dates, study drug administration, primary evaluation criteria, nature of adverse events with date of start and related treatment (Further details are available in the Investigator Operating Manual).

Criteria of efficacy evaluation for which the CRF is considered as source document will be stated in the Investigator Operating Manual.

The investigator will permit study-related monitoring, audit(s), and regulatory inspection(s), with a direct access to all the required source documents each time it is necessary provided that patient confidentiality is protected.

Source documents should be preserved for the maximum period of time permitted by local requirements.

14.2 Periodic monitoring

The monitor will contact and visit the investigator periodically to evaluate study progress and protocol compliance. For this study, the average frequency of the monitoring visits is intended to be approximately every 4 to 6 weeks with the first visit occurring as soon as possible after the first patient inclusion. Intervals may be adjusted according to patient accruals, protocol changes or site performance.

The investigator and any study staff member will co-operate with the monitor to ensure that any problems are resolved.

14.3 Audit and inspection

After appropriate notification, the investigator will make all study-related source data and documents available to a quality assurance auditor mandated by Transgene, or to domestic or foreign regulatory inspectors. The main purposes of an audit or inspection are to confirm that

the rights and well-being of the patients have been adequately protected, and that all data relevant for the evaluation of the study drug have been processed and reported in compliance with GCP and applicable regulatory requirements.

15 DATA HANDLING AND RECORD KEEPING

15.1 Investigators information

The investigator will be kept informed on an ongoing basis of important information that relates to the study procedures including safety issues.

The site personnel, e.g. residents, nurses, laboratory technicians and any other personnel providing care to the patients or handling biological specimen will be informed on study procedures and receive safety instructions.

15.2 Case report forms

For each patient randomized/included, a CRF must be completed, as far as possible in English, by the investigator or designee and signed by the investigator. This does not apply to records for those patients not randomized/included. If a patient is withdrawn from the study, the reason must be noted in the CRF. If a patient is withdrawn from the study because of a treatment-limiting adverse event, thorough efforts should be made to clearly document the outcome.

Data reported in the CRF will be verified against source documents before being collected.

CRFs will consist of no carbon required (NCR) paper. They should be completed in a neat, legible manner to ensure adequate interpretation of data. A black ballpoint pen should be used to ensure clarity of all reproduced CRFs.

15.3 Changes to case report form data

Errors occurring in CRFs will be crossed out with a single line without obscuring the initial entry, the correction will be written alongside the initial entry, and the change will be initialed and dated by the investigator or designee. If not obvious, the reason of the change will be given on the additional comments pages. Correction fluid or any other means of obliteration of entries must not be used. Any correction made on the original CRF page must also appear clearly on all copies of CRF pages.

A query form that is signed by the investigator or designee will be used to document any changes to CRF data which are required after the original CRF has been retrieved from the site by the monitor.

15.4 Provision of additional information

If requested by the sponsor, additional data (duly anonymized) relating to the study, including copies of relevant source documents, will be provided by the investigator. This may be necessary when CRFs are illegible or when errors in data transcription are encountered. It

may be necessary to access the complete source documents, provided that the patients' confidentiality is protected in accordance with local regulations.

16 REPORTING AND PUBLICATION

16.1 Clinical study report

All relevant data will be reported in a study report which will be prepared by Transgene/CRO and submitted for comments and signature to the coordinating / principal investigator. The final report is used by the sponsor for regulatory purposes according to local regulation.

16.2 Confidentiality of study data

Any confidential information relating to the study drug or the study, including any data and results from the study is the property of Transgene. Documents are supplied to the investigators under conditions of strict confidentiality. Neither the investigator nor any person working on his/her behalf may disclosure any of the information therein without having obtained prior written consent from Transgene.

16.3 Publication policy

The results of this study may be published or presented at scientific meetings. If this is envisaged, the investigator agrees to submit all manuscripts or abstracts to Transgene prior to scientific meeting or journal submission. This allows the sponsor to protect proprietary information and to provide comments based on information from other studies that may not yet be available to the investigator. The publication rules will follow the recruitment rate, then Transgene representatives. Principal Investigator as first or last name.

In accordance with consistent editorial practice, Transgene supports publication of multicenter studies in their entirety and not as individual center data unless ancillary study/data. A publication in which the contribution of the sponsor's personnel exceeded that of conventional monitoring will be considered as a joint publication by the investigator and this person.

17 ARCHIVING

17.1 Investigator site file

In accordance with the ICH GCP standards, the investigator is responsible for on-site storage and maintenance of all records pertaining to the study.

This documentation should be kept by the investigator at least 2 years following the date the last marketing application is approved for the study drug in the indication being investigated in this study or if no application is to be filed or if the application is not approved for such indication, until 2 years after the investigation is discontinued. If longer archiving period is required, the local law/regulation must be followed. Transgene will inform the investigator when documents may be destroyed.

Not study site document may be destroyed without prior written agreement between the investigator and the sponsor. The sponsor must be notified if the investigator assigns the study documentation to another party or moves it to another location.

If the investigator cannot guarantee this archiving requirement on site for any or all of the documents, special arrangements must be made between the investigator and Transgene to store the documents in a sealed container off-site so they can be returned sealed to the investigator in case of an audit/inspection.

17.2 Trial master file

Transgene will archive the trial master file (TMF) in accordance with GCP and applicable regulatory requirements, and will inform the investigator when the archiving of the study documentation is no longer required.

18 REFERENCES

Behbehani A.M., The smallpox story: life and death of an old disease. *Microbiological reviews* (1983), 47, 455-509.

Bubenik J., IL-2 and gene therapy of cancer - Review. *International Journal of Oncology* (1993), 2, (6) 1049-1052.

Burchell J., Gendler S., and Taylor-Papadimitriou J., Developpment and characterization of breast cancer reactive monoclonal antibodies directed to the core protein of the human milk mucin. *Cancer Research* (1987), 47, 5476-5482.

Carroll M.W. and Moss B., Host range and cytopathogenicity of the highly attenuated MVA strain of vaccinia virus: propagation and generation of recombinant viruses in a nonhuman mammalian cell line. *Virology* (1997), 238, 198.

Cella D.F., Bonomi A.E., Lloyd S.R., Tulsky D.S., Kaplan E., and Bonomi P., Reliability and validity of the Functional Assessment of Cancer Therapy-Lung (FACT-L) quality of life instrument. *Lung Cancer* (1995), 12, (3) 199-220.

Delbaldo C., Michiels S., Syz N., Soria J.C., Le Chevalier T., and Pignon J.P., Benefits of adding a drug to a single-agent or a 2-agent chemotherapy regimen in advanced non-small-cell lung cancer: a meta-analysis. *JAMA* (2004), 292, (4) 470-84.

Devine P., Warren J.A., Ward B.G., McKenzie I.F., and Layton G.T., Glycosylation and the exposure of tumor -associated epitopes on mucins. *Journal of Tumor Marker Oncology* (1990), 5, (1) 11-26.

Fleming T.R., One-sample multiple testing procedure for phase II clinical trials. *Biometrics* (1982), 38, (1) 143-51.

Foa R., Guarini A., and Gansbacher B., IL2 treatment for cancer: from biology to gene therapy. *British Journal of Cancer* (1992), 66, 992-998.

Freedman LS, White SJ. On the use of Pocock and Simon’s method for balancing treatment numbers over prognostic factors in the controlled clinical trial. *Biometrics* (1976) 32: 691-694.

Fujita K., Denda K., Yamamoto M., Matsumoto T., Fujime M., and Irimura T., Expression of MUC1 mucins inversely correlated with post-surgical survival of renal cell carcinoma patients. *British Journal of Cancer* (1999), 80, (1-2) 301-308.

Gansbacher B.K., Zier B., Daniels K., Kronin R., Bannerji E., and Gilboa E., Interleukin 2 gene transfer into tumor cells abrogates tumorigenicity and induces protective immunity. *Journal of Experimental Medicine* (1990), 172, 1217-1224.

Ginsberg R.J., Vokes E.E., and Rosenweig K., Non-small cell lung cancer. (2001), 6, 6, DeVita V Jr, Hellman S, Rosenberg SA, Philadelphia, PA, Lippincott-Raven, 925-983.

Govindan R., Cetuximab in advanced non-small cell lung cancer. *Clinical Cancer Research* (2004), 10, (12 Pt 2) 4241s-4244s.

Hanna N., Shepherd F.A., Fossella F.V., Pereira J.R., De Marinis F., von Pawel J., Gatzemeier U., Tsao T.C., Pless M., Muller T., Lim H.L. , Desch C., Szondy K., Gervais R., Shaharyar:, Manegold C., Paul S., Paoletti P., Einhorn L., and Bunn P.A.Jr., Randomized phase III trial of pemetrexed versus docetaxel in patients with non-small-cell lung cancer previously treated with chemotherapy. *Journal of Clinical Oncology* (2004, May 1), 22, (9) 1589-97.

Hareuveni M., Tsarfaty I., Zaretsky J., Kotkes P., Horev J., Zrihan S., Weiss M., Green S., Lathe R., Keydar I., and Wreschner D.H., A transcribed gene, containing a variable number of tandem repeats, codes for a human epithelial tumor antigen. *European Journal of Biochemistry* (1990), 189, 475-486.

Ho S.B., Niehans G.A., Lyftog C., Yan P., Cherwitz D., Gum E., Dahiya R., and Kim Y., Heterogeneity of mucin gene expression in normal and neoplasic tissues. *Cancer Research* (1993), 53, 641-651.

Jemal A., Ward E., Wu X., Martin H.J., McLaughlin C.C., and Thun M.J., Geographic patterns of prostate cancer mortality and variations in access to medical care in the United States. *Cancer Epidemiology, Biomarkers & Prevention* (2005), 14, (3) 590-595.

Johnson D.H., Fehrenbacher L., Novotny, W.F., Herbst R.S., Nemunaitis J.J., Jablons D.M., Langer C.J., DeVore R.F.3., Gaudreault J., Damico L.A., Holmgren E., and Kabbinavar F., Randomized phase II trial comparing bevacizumab plus carboplatin and paclitaxel with carboplatin and paclitaxel alone in previously untreated locally advanced or metastatic non-small-cell lung cancer. *Journal of Clinical Oncology* (2004), 22, (11) 2184-91.

Kaplan G., Cohn Z.A., and Smith K.A., Rational immunotherapy with interleukin 2. *Biotechnology (N.Y.)* (1992), 10, (2) 157-62.

Konrad M.W., Hemstreet G., and Hersh E.M., Pharmacokinetics of recombinant interleukin 2 in humans. *Cancer Research* (1990), 50, 2009-2017.

Langer C.J., Emerging role of epidermal growth factor receptor inhibition in therapy for advanced malignancy: focus on NSCLC. *International Journal of Radiation Oncology, Biology, Physics* (2004), 58, (3) 991-1002.

Layton G.T., Devine P.L., and Warren P.L., Monoclonal antibodies reactive with the breast carcino-associated mucin core protein repeat sequence peptide also recognise the ovarian carcinoma-associated sebaceous gland antigen. *Tumour Biology* (1990), 11, 274-286.

Mahnel H. and Mayr A., Erfahrung bei der Schutzimpfung gegen Orthopocken von Mensch und Tier mit dem Impstamm MVA. *Berliner und Munchener Tierarztliche Wochenschrift* (1994), 107, 253-256.

Mayr A., Hochstein-Mintzel V., and Stickl H., Abstammung, Eigenschaften und Verwendung des attenuirten Vaccinia-Stammes MVA. *Infection* (1975), 3, (1) 6-14.

Mayr A., Stickl H., Müller H.K., Danner K., and Singer H., Der Pockenimpfstamm MVA: Marker, genetische Struktur, Erfahrung mit der parenteralen Schutzimpfung und Verhalten im abwehrgeschwächten Organismus. *Zentralblatt fur Bakteriologie, Mikrobiologie und Hygiene.1. Abt. Originale. B: Umwelthygiene. Krankenhaushygiene. Arbeitshygiene. Praventive Medizin.* (1978), 167, 375-390.

NCLC, Chemotherapy in non-small cell lung cancer: a meta-analysis using updated data on individual patients from 52 randomised clinical trials. Non-small Cell Lung Cancer Collaborative Group. *BMJ* (1995, Oct 7), 311, (7010) 899-909.

Parkin D.M., International variation. *Oncogene* (2004), 23, (38) 6329-40.

Peat N., Gendler S.J., Lalani E.N., Duhig T., and Taylor-Papadimitriou J., Tissue-specific expression of a human polymorphic epithelial mucin. *Cancer Research* (1992), 52, 1954-1960.

Schmidt I., *Detection by PCR of recombinant vaccinia virus TG4010 in patients included in Phase I clinical trial TG4010.02*, (Study Report), 70/34/6008/01, Paris, (2000).

Schmidt I., *Detection by PCR of recombinant vaccinia virus TG4010 in patients included in Phase I clinical trial TG4010.01*, (Study Report), 70/34/6008/02, Paris, (2001).

Stickl H., Hochstein-Mintzel V., Mayr A., Huber H.C., Schefer H., and Holzner A., MVA-Stufenimpfung gegen Pocken. Klinische Erprobung des attenuierten Pocken-Lebendimpfstoffes, Stammes MVA. *Deutsche Medizinische Wochenschrift* (1974), 99, 2386-2392.

Sutter G. and Moss B., Nonreplicating vaccinia vector efficiently expresses recombinant genes. *Proceedings of the National Academy of Sciences of the USA* (1992), 89, 10847-10851.

Sutter G., Wyatt L.S., Foley P.L., Bennink J.R., and Moss B., A recombinant vector derived from the host range-restricted and highly attenuated MVA strain of vaccinia virus stimulates protective immunity in mice to influenza virus. *Vaccine* (1994), 12, (11) 1032-40.

19 APPENDICES

Appendix 1a: Study flow-chart arm 1
Appendix 1b: Study flow-chart arm 2
Appendix 2: Performance status criteria (ECOG)

APPENDIX 1a

FLOW-CHART- Arm 1: TG4010 + Chemotherapy

		Baseline	First 6-week period						Second 6-week period				Subsequent every 6-week periods				End of study §
**Study days**		**-21/0**	**1**	**8**	**15**	**22**	**29**	**36**	**43**	**50**	**64**	**71**	**85 127…**	**92**	**106**	**113**	
**Injection TG4010**			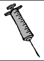	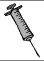	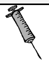 @	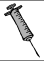	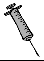	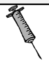 @	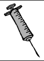		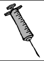		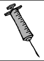		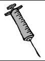		
**Chemotherapy (up to 6 cycles of 3 weeks)**	**Gemcitabine**		X	X		X	X		X	X	X	X	X	X	X	X	
	**Cisplatin**		X			X			X		X		X		X		
**Clinical evaluation**		X	X	X	X	X	X	X	X	X	X	X	X	X	X	X	X
**Safety assessment**			X	X	X	X	X	X	X	X	X	X	X	X	X	X	X
**Quality of life assessment (FACT-L)**		X							X				X				X
**Tumor evaluation***		X							X				X				
**Tumor markers** CA15.3, CEA, CA19.9, CA125, CYFRA21-1**		X	X			X			X		X		X		X		X
**Hematology**		X	X	X	X	X	X	X	X	X	X	X	X	X	X	X	X
**Laboratory evaluation**		X	X			X			X		X		X		X		X
**Auto-immunity parameters**		X							X				X				X
**Immunology evaluation / Proteomic**		X							X				X¤				
**ECG, chest X-ray, cerebral CT scan/MRI**		X															
**Serology (HIV, HCV and HBV) and βHCG**		X															
**HLA typing (MHC class I)**		X															
**Histological diagnosis / MUC1 staining**		X															

*Results must be available prior to the visit
**After baseline, only the initially elevated markers will be tested
@ The injection of TG4010 on days 15 and 36 will be done only if the hematological function is adequate
¤ Only at day 85
§ Upon withdrawal from study and at least 4 weeks after the last TG4010/chemotherapy injection

Assessments will be done prior to treatment.

APPENDIX 1b

FLOW-CHART- Arm 2: Chemotherapy alone

		Baseline	First 6-week period						Second 6-week period				Subsequent every 6-week periods				End of study §
**Study days**		**-21/0**	**1**	**8**	**15**	**22**	**29**	**36**	**43**	**50**	**64**	**71**	**85 127…**	**92**	**106**	**113**	
**Chemotherapy (up to 6 cycles of 3 weeks)**	**Gemcitabine**		X	X		X	X		X	X	X	X	X	X	X	X	
	**Cisplatin**		X			X			X		X		X		X		
**Clinical evaluation**		X	X	X		X	X		X	X	X	X	X	X	X	X	X
**Safety assessment**			X	X		X	X		X	X	X	X	X	X	X	X	X
**Quality of life assessment (FACT-L)**		X							X				X				X
**Tumor evaluation***		X							X				X				
**Tumor markers** CA15.3, CEA, CA19.9, CA125, CYFRA21-1**		X	X			X			X		X		X		X		X
**Hematology**		X	X	X		X	X		X	X	X	X	X	X	X	X	X
**Laboratory evaluation**		X	X			X			X		X		X		X		X
**Auto-immunity parameters**		X							X				X				X
**Immunology evaluation / Proteomic**		X							X				X[¤]				
**ECG, chest X-ray, cerebral CT scan/MRI**		X															
**Serology (HIV, HCV and HBV) and βHCG**		X															
**HLA typing (MHC class I)**		X															
**Histological diagnosis / MUC1 staining**		X															

*Results must be available prior to the visit

**After baseline, only the initially elevated markers will be tested

[¤] Only at day 85

§ Upon withdrawal from study and at least 4 weeks after the last chemotherapy injection

Assessments will be done prior to treatment.

APPENDIX 2

PERFORMANCE STATUS (ECOG) SCALE

0. Fully active, able to carry on all pre-disease performance without restriction.

1. Restricted in physically strenuous activity but ambulatory and able to carry out work of a light or sedentary nature, e.g. light house work, office work.

2. Ambulatory and capable of self care but unable to carry out any work activities. Up and about more than 50% of waking hours.

3. Capable of only limited self-care, confined to bed or chair more than 50% of waking hours.

4. Completely disabled. Cannot carry out any self-care. Totally confined to bed or chair.
